# Effects of exercise on bone metabolism in postmenopausal women: a systematic review and meta-analysis of randomized controlled trials

**DOI:** 10.3389/fendo.2025.1597046

**Published:** 2025-09-15

**Authors:** Wenhua Zhang, Xun Li, Qiang He, Xiaoqiang Wang

**Affiliations:** ^1^ School of Graduate Education, Shandong Sport University, Jinan, Shandong, China; ^2^ School of Sport and Health, Shandong Sport University, Jinan, Shandong, China; ^3^ School of Physical Education, Shandong University, Jinan, Shandong, China

**Keywords:** exercise, postmenopausal women, bone metabolism, osteoporosis, osteopenia, randomized controlled trial, meta-analysis

## Abstract

**Objective:**

To assess the impact of exercise on bone metabolism in postmenopausal women through meta-analysis, and to offer evidence-based guidance for preventing and managing osteoporosis in this population.

**Methods:**

We searched PubMed, Embase, and other databases using keywords such as “exercise,” “postmenopausal women,” and “bone metabolism” to identify randomized controlled trials published up to 2024 on the effect of exercise on bone metabolism in postmenopausal women. Studies were selected according to predefined inclusion and exclusion criteria. Data were analyzed using Review Manager 5.4 and Stata17. Study quality was assessed with the Cochrane risk-of-bias tool. Effect sizes were pooled as standardized mean differences(*SMDs*)with 95% confidence intervals(*CIs*), and heterogeneity was evaluated with the I²statistic. A fixed-effects model was used when *I²*≤50%; otherwise, a random-effects model was applied. The overall evidence certainty was rated using the Grading of recommendations assessment, development, and evaluation(GRADE)system.

**Results:**

A meta-analysis of 24 studies(1067 subjects total)showed that exercise significantly elevated the levels of alkaline phosphatase(ALP)(*SMD* = 0.49, 95%*CI*: 0.21-0.77, *P* = 0.0006), N-terminal propeptide of type I procollagen(P1NP)(*SMD* = 0.62, 95% *CI*: 0.24 to 1.01, *P* = 0.002)and osteocalcin(OC)(*SMD* = 0.21, 95% *CI*: 0.05 to 0.37, *P* = 0.01); exercise significantly reduced the levels of parathyroid hormone(PTH)(*SMD*=-0.51, 95% *CI*: -0.77 to -0.25, *P* = 0.0001)and type I collagen cross-linked C-terminal peptide(CTX)(*SMD*=-0.32, 95% *CI*: -0.51to-0.12, *P* = 0.001). Subgroup analyses showed that aerobic exercise(*SMD*=-0.35, 95% *CI*: -0.65 to -0.06, *P* = 0.02) significantly reduced CTX levels, while both aerobic exercise(*SMD* = 0.23, 95% *CI*: 0.01 to 0.44, *P* = 0.04)and resistance exercise(*SMD* = 0.65, 95% *CI*: 0.10 to 1.20, *P* = 0.02)significantly increased OC levels. Exercise interventions lasting ≤6 months(*SMD*=-0.45, 95% *CI*: -0.72 to -0.18, *P* = 0.001)and sessions of ≤60 min(*SMD*=-0.48, 95% *CI*: -0.80 to -0.17, *P* = 0.003)both significantly reduced CTX levels, exercise interventions lasting ≤6 months(*SMD* = 0.35, 95% *CI*: 0.13 to 0.57, *P* = 0.002)and sessions of ≤60 min(*SMD* = 0.20, 95% *CI*: 0.01 to 0.39, *P* = 0.04)can significantly both increase OC levels.

**Conclusion:**

Exercise significantly improves bone metabolism in postmenopausal women by reducing bone resorption and promoting bone formation. Aerobic exercise lowers CTX levels, while both aerobic and resistance exercise increase OC levels. Short-term (≤6 months) and moderate-length (≤60 minutes/session) interventions are particularly effective. However, more high-quality randomized controlled trials are needed to confirm these benefits.

**Systematic review registration:**

https://www.crd.york.ac.uk/, identifier CRD42024610810.

## 1 Introduction

Osteoporosis is a systemic metabolic bone disease whose main risk is fragility fracture and is recognized as a disease that seriously affects the public health of society (1). Numerous studies have confirmed that postmenopausal women are at high risk of developing osteoporosis, with approximately 200 million women worldwide suffering from osteoporosis after menopause. After menopause, there is destruction of the trabecular structure of the bone, which in turn leads to an increase in the brittleness of the bone and a decrease in the mechanical strength of the bone, which in turn increases the risk of fracture (2). With increasing age, decreased physical activity, insufficient calcium intake, decreased absorption, and decreased vitamin conversion can make bone calcium highly susceptible to absorption and migration (3). Therefore, it is of great practical significance to explore exercise to improve bone metabolism and prevent osteoporosis in postmenopausal women.

Previous studies have demonstrated that exercise improves bone metabolism in postmenopausal women, thereby effectively preventing and treating osteoporosis (4, 5). The effect of exercise on bone metabolism in postmenopausal women has been a hot research topic. Exercise can improve bone metabolism by stimulating the secretion of growth factors by bone cells, promoting blood circulation in the skeletal system, and accelerating the absorption and metabolism of nutrients (6). Additionally exercise can stimulate adaptive changes in the skeletal system by increasing muscle loading, increasing bone density and bone strength and thus improving bone health (7).

The effects of exercise on bone health in postmenopausal women have been reviewed with a focus on bone mineral density (BMD) (8–10), which is the most commonly used metric for assessing bone health and reflects a relatively static bone mass status (11). BMD does not fully reflect bone metabolism, and in order to overcome this limitation and to understand the dynamic response to bone remodeling with exercise, bone metabolism markers are therefore used as dynamic indicators to evaluate bone remodeling (11). In addition, as bone metabolism increases with age, the potential clinical application of these markers could assess fracture risk and measure bone health in postmenopausal women (12). This article reviews the relevant studies on the changes in various bone metabolism indicators in postmenopausal women after various interventions such as aerobic exercise, resistance exercise, impact exercise, Tai Chi, whole-body vibration training, and combined exercise. Meta analysis is conducted on the outcome indicators to compare the effects of exercise on bone metabolism, providing a theoretical basis for postmenopausal women to exercise scientifically to prevent and treat osteoporotic fractures.

## 2 Materials and methods

### 2.1 Protocol and registration

This comprehensive systematic review and meta-analysis followed the guidelines outlined in the Preferred Reporting Items for Systematic Reviews and Meta-Analyses (PRISMA) (13) (**Supplementary Table S1**). This study was registered with the PROSPERO platform under the registration number: CRD42024610810 (https://www.crd.york.ac.uk/).

### 2.2 Search strategy

The first author conducted the search from November 6 to 8, 2024. The databases Retrieved November 2024 by the first author. PubMed, Embase, Cochrane Library, Web of Science, Scopus and Google Scholar were searched. Randomized controlled trials (RCTs) on the effects of aerobic and resistance exercise on bone metabolism in middle-aged and elderly people were searched in each database. The search terms included “Exercise, physical exercise, postmenopausal women, osteoporosis, low bone mass, bone metabolism, randomized controlled trial”. To identify more potential studies, we manually searched gray literature, reference lists of identified studies, and relevant registration website (ClinicalTrials.gov) and consulted experts in this field. however, due to the lack of standardized peer-review processes and limited accessibility of detailed data, we decided to exclude gray literature from our analysis. The full search strategies for all databases are shown in **Supplementary Table S2**.

### 2.3 Inclusion and exclusion criteria for the studies

Inclusion criteria: Inclusion criteria: (i) Study type: RCTs on the effects of exercise on bone metabolism in middle-aged and older adults that have been published in various databases from the time of their construction to November 6, 2024. Only peer-reviewed articles published in English were included to ensure methodological quality and accessibility of data; (ii) Study subjects: postmenopausal women aged ≥45 years. In this study, “postmenopausal” was defined as a serum follicle-stimulating hormone (FSH) level >25–30 IU/L, natural amenorrhea for >12 months, or explicit mention in the literature that the participants were in the postmenopausal stage. (iii) Intervention: any form of physical exercise (which refers to the cultural activities in which participants are physically active through the importance of physical exercise with the purpose of strengthening their physical fitness and improving their health), and the intervention group was based on Physical exercise is the focus of the intervention group, and there is no requirement for the exercise mode and load volume. Control measures: the control group only carries out daily life or original physical exercise and does not receive additional exercise intervention. If the control group receives conventional treatment, the experimental group should adopt conventional treatment + exercise intervention at the same time. (iv) Out-come indicators: serum phosphorus, serum calcium, osteocalcin (OC), 25-hydroxyvitamin D (25(OH)D), alkaline phosphatase (ALP), parathyroid hormone (PTH), type I collagen carboxy-terminal peptide (CTX), N-terminal propeptide of type I procollagen (P1NP).

Exclusion criteria: (i) Duplicated published literature; (ii) Non-RCT; (iii) Study subjects were not postmenopausal women; (iv) Interventions did not meet the inclusion criteria and controls did not meet the inclusion criteria; and (v) Outcome metrics did not meet the outcome metrics associated with the inclusion criteria; (vi) Unpublished studies, conference abstracts, and grey literature were excluded due to the lack of complete methodological details and peer review, which may affect data reliability.

### 2.4 Literature screening and data extraction

The retrieved literature was imported into Endnote 20 software and duplicates were removed from it. Two researchers then screened the literature and extracted information based on established inclusion and exclusion criteria. If disagreements were encountered, they were discussed and resolved with a third researcher. Characteristics of included studies included authors, year of publication, country, sample size, and age (**Table 1**), and characteristics of included interventions included type of exercise, frequency of exercise, exercise period, duration of a single exercise session, control exercise status, and outcome indicators (**Table 2**). Bone metabolism marker data were extracted as post-intervention mean and standard deviation (*SD*).

**Table 1:** Research characteristics.

Author (Year)	Country and area	Subject type	Sample size (*n*)		Age		Bone status (diagnostic criteria, measurement sites, and measurement tools)
			EG	CG	EG	CG	
Hatori (16) (1993)	Japan	Post-menopausal women	9/12	12	MG: 58 ± 5 HG: 56 ± 4	58 ± 8	Healthy, no osteoporosis
Nelson (17) (1994)	United States of America	Post-menopausal women	20	18	61.4 ± 6.9	58.9 ± 8.1	Healthy, no osteoporosis
Iwamoto (18) (2001)	Japan	Post-menopausal women	8	20	65.3 ± 4.7	64.9 ± 5.7	Osteoporosis: BMD more than 30% below the young adult mean (in accordance with the Japanese diagnostic guidelines for osteoporosis) Measurement site: Lumbar spine (L2–L4) BMD Measurement tool: DXA
Ay (21) (2003)	Turkish	Post-menopausal women	23	23	54.28 ± 6.08	55.11 ± 5.32	Osteopenia or osteoporosis: BUA T-score ≤ -1 Measurement site: calcaneus Measurement tool: QUS
Wu (19) (2005)	Japan	Post-menopausal women	31	33	54.4 ± 2.9	53.8 ± 2.9	Healthy, no osteoporosis
Wu (20) (2006)	Japan	Post-menopausal women	31	33	54.4 ± 2.9	53.8 ± 2.9	Healthy, no osteoporosis
Shen (38) (2010)	United States of America	Post-menopausal women	44	42	58.3 ± 7.7	57.6 ± 7.5	Osteopenia: -2.5 < T-score < -1.0 Measurement sites: Lumbar spine or hip BMD Measurement tool: DXA
Tartibian (22) (2011)	Iran	Post-menopausal women	20	18	61.4 ± 6.9	58.9 ± 8.1	Healthy, no osteoporosis
Wayne (37) (2012)	United States of America	Post-menopausal women	43/26	43	58.8 ± 5.5/ 59.1 ± 4.9	60.4 ± 5.3	Osteopenia: -2.5 < T-score < -1.0 Measurement sites: lumbar spine (L1–L4), femoral neck, and total hip BMD Measurement tool: DXA
Bergström (23) (2012)	Sweden	Post-menopausal women	48	44	58.9 ± 4.3	59.6 ± 3.6	Osteopenia: -2.5 < T-score < -1.0 Measurement sites: hip or spine BMD Measurement tool: DXA
Baset (24) (2013)	Turkey	Post-menopausal women	11	12	SG: 55.9 ± 4.9 HIG: 55.6 ± 2.9	56.2 ± 4.0	Osteopenia: -2.5 < T-score < -1.0 Measurement sites: Lumbar spine (L_1_-L_4_) or femoral neck BMD Measurement tool: DXA
Mosti (25) (2013)	Norway	Post-menopausal women	8	8	61.9 ± 5.0	66.7 ± 7.4	Osteoporosis: T-score ≤ -2.5; Osteopenia: -2.5 < T-score < -1.0 Measurement sites: Lumbar spine (L1–L4), femoral neck, and total hip BMD Measurement tool: DXA
Roghani (26) (2013)	Iran	Post-menopausal women	8/9	10	45-65		Osteoporosis: T-score ≤ -2.5 Measurement sites: lumbar spine or hip BMD Measurement tool: DXA
Pernambuco (27) (2013)	Brazil	Post-menopausal women	36	31	66.8 ± 4.2		Normal bone mass: T-score ≥ -1.0; osteoporosis: BMD T-score ≤ -2.5; osteopenia: -2.5 < T-score < -1.0 Measurement sites: lumbar spine (L_2_–L_4_) and total hip BMD Measurement tool: DXA
Moreira (28) (2014)	Japan	Post-menopausal women	59	41	58.6 ± 6.71	59.3 ± 6.07	Normal bone mass: T-score ≥ -1.0; Osteopenia: -2.5 < T-score < -1.0; Osteoporosis: T-score ≤ -2.5 Measurement sites: lumbar spine (L_1_–L_4_), femoral neck, trochanter, total hip, and whole-body BMD Measurement tool: DXA
Wen (6) (2017)	China, Taiwan	Post-menopausal women	24	24	57.5 ± 3.5	58.8 ± 3.2	Osteopenia: T-score ≤ -1.0; Osteoporosis: T-score ≤ -2.5 Measurement sites: lumbar spine, hip, and whole-body BMD Measurement tool: DXA
Baker (29) (2018)	Australia	Post-menopausal women	14	17	61.6 ± 9.2	61.6 ± 7.8	Only 4 participants (12.9%) met the criteria for osteoporosis (T-score ≤ −2.5)
Sen (30) (2020)	Turkey	Post-menopausal women	15	18	WG: 55.0 ± 4.6 HIG: 53.1 ± 4.4	54.5 ± 6.0	The BMD T-score at the lumbar spine (L_1_–L_4_ or L_2_–L_4_) and/or femoral neck and total hip ranges from -2.0 to -3.0, meeting the diagnostic criteria for osteopenia or osteoporosis (according to WHO standards: T-score ≤ -2.5 indicates osteoporosis, and -1.0 to -2.5 indicates low bone mass/osteopenia)
Linero (31) (2021)	South Korea	Post-menopausal women	7/6/6	5	MHIRT: 56.43 ± 0.72 LIBFR: 55.71 ± 0.52 LIRT: 56.50 ± 0.99	56.83 ± 0.70	Osteoporosis: T-score ≤ -2.5; Osteopenia: -2.5 < T-score < -1.0 Measurement sites: lumbar spine or femoral neck BMD Measurement tool: DXA
Pereira (32) (2021)	Portugal	Post-menopausal women	41	26	67.3 ± 6.5	69.9 ± 5.4	Healthy, no osteoporosis
Kim (33) (2022)	South Korea	Post-menopausal women	14	15	81.14 ± 3.98	80.80 ± 2.37	NR
Zaravar (34) (2024)	Iran	Post-menopausal women	7	6	65.100 ± 3.478	66.400 ± 3.238	The mean baseline BMD (0.517–0.585 g/cm²) was significantly lower than that of healthy young adults. Based on the baseline BMD values, the participants were diagnosed with osteopenia or osteoporosis
Guzel (35) (2024)	Turkey	Post-menopausal women	12	12	55.67 ± 3.44	54.42 ± 4.01	Healthy, no osteoporosis
Pasa (36) (2024)	Indonesia	Post-menopausal women	14	14	45-69		According to the guidelines of the World Health Organization (WHO) and the International Society for Clinical Densitometry (ISCD, 2023), BMD T-scores are classified as follows: Normal bone mass: T-score ≥ -1; Osteopenia: -2.5 < T-score < -1; Osteoporosis: T-score ≤ -2.5 Measurement device: Sonost 3000 densitometer Measurement site: calcaneus

EG, exercise group; CG, control group; HG, high intensity group; MG, moderate intensity group; WG, whole-body vibration (WBV); SG, strength training group; HIG, high impact training group; QUS, quantitative ultrasound; BUA, broadband ultrasound attenuation; DXA, dual-energy X-ray absorptiometry; BMD, bone mineral density; MHIRT, moderate to high-intensity resistance training; LIBFR, low-intensity resistance training with blood flow restriction; LIRT, low-intensity resistance training.

**Table 2:** Characterization of research interventions.

Athor	Interventions	Frequency of intervention	Intervention cycle	Duration of one intervention	Exercise status of the control group during the trial	Outcome measures (units, baseline ranges, measurement methods)
Hatori (16)	Walking	3 times/week	7 months	30 minutes	Not undertaking a specific exercise program	Baseline ranges: Ca (mg/dL): 9.0 ± 0.2 (CG), 9.2 ± 0.4 (MG), 9.4 ± 0.5 (HG) P (ng/mL): 3.6 ± 0.3 (CG), 3.5 ± 0.3 (MG), 3.7 ± 0.5 (HG) OC (ng/mL): 11.8 ± 2.6 (CG), 12.0 ± 2.3 (MG), 11.6 ± 2.4 (HG) ALP (U/L): 167 ± 29 (CG), 177 ± 30 (MG), 196 ± 37 (HG) Measurement methods: OC was measured by RIA; Ca, P, and ALP were measured using automated methods
Nelson (17)	High-intensity strength training	2 times/week	1year	45 minutes	No strength training program	Baseline ranges: OC (nmol/L): 1.163 ± 0.224 (CG), 1.094 ± 0.245 (EG) 25(OH)D (nmol/L): 79.8 ± 27.5 (CG), 68.3 ± 25.2 (EG) PTH (pmol/L): 2.853 ± 0.851 (CG), 2.855 ± 1.011 (EG) Measurement methods: OC and PTH were measured using IRMA, and 25(OH)D was measured using Competitive Protein-Binding Analysis
Iwamoto (18)	Brisk walking and gymnastic exercises	NR	2 years	NR	Daily activities only, no additional exercise interventions	Baseline ranges: Ca (mg/dl): 9.4 ± 0.4 (EG), 9.3 ± 0.4 (CG) P (mg/dl): 3.6 ± 0.4 (EG), 3.5 ± 0.4 (CG) ALP (IU/l): 214 ± 69 (EG), 216 ± 52 (CG) Measurement method: Ca, P, and ALP were measured using standard automated laboratory techniques
Ay (21)	Aquatic aerobics exercises	3 times/week	6 months	45 minutes	The control group was asked to maintain their sedentary lifestyle throughout the study period	Baseline ranges: PTH (pg/mL): 72.68 ± 47.01 (EG), 68.89 ± 25.34 (CG). Measurement method: Iodine-125 RIA
Wu (19)	Walking	3 times/week	24 weeks	45 minutes	No additional exercise intervention	Baseline ranges: OC (ng/mL): 9.50 ± 2.42 (EG), 9.23 ± 2.09 (CG) Measurement method: Sandwich enzyme immunoassay using polyclonal antibodies
Wu (20)	Walking	3 times/week	1 year	45 minutes	No additional exercise intervention	Baseline ranges: OC (ng/mL): 9.50 ± 2.42 (EG), 9.23 ± 2.09 (CG) Measurement method: Sandwich enzyme immunoassay using polyclonal antibodies
Shen (38)	Tai Chi	3 times/week	24 weeks	60 minutes	Placebo, no additional exercise intervention	Baseline ranges: P (mg/dl): 3.7 ± 0.5 (EG and CG), 3.7 ± 0.5 (CG) Ca (mg/dl): 9.4 ± 0.2 (EG), 9.4 ± 0.4 (CG) ALP (U/L): 81.6 ± 20.1 (EG), 75.3 ± 18.6 (CG) Detection method: NR
Tartibian (22)	Walking and running exercises	3–4 times/week	12/24 weeks	25–30 minutes	Daily activities only, no additional exercise interventions	Baseline ranges: P (mg/dL): 3.8 ± 0.5 (EG), 3.6 ± 0.6 (CG) Ca (mg/dL): 9.5 ± 0.7 (EG), 9.3 ± 0.7 (CG) CTX (ng/mL): 0.5 ± 0.1 (EG and CG) 25(OH)D (pg/mL): 41.5 ± 21.9 (EG), 42.3 ± 20.6 (CG) PTH (pg/mL): 92.5 ± 46.6 (EG), 94.9 ± 46.6 (CG) OC (ng/mL): 25.8 ± 8.4 (EG), 24.4 ± 7.7 (CG) Measurement methods: CTX and OC were measured using ELISA, 25(OH)D by Radioreceptor Assay, PTH by Electrochemiluminescent Method P, Ca: NR
Wayne (37)	Tai Chi	4 times/week	9 mouths	90 minutes	No designated athletic training, only routine medical care	Baseline ranges: CTX (ng/mL): 0.554 ± 0.259 (Randomized to Tai Chi), 0.594 ± 0.30 (Per Protocol Tai Chi), 0.603 ± 0.231 (Randomized to Usual Care) OC (ng/mL): 16.29 ± 6.01 (Randomized to Tai Chi), 15.52 ± 4.94 (Per Protocol Tai Chi), 17.11 ± 7.02 (Randomized to Usual Care) Measurement methods: CTX was measured using ELISA, and OC was RIA
Bergström (23)	Aerobic exercise	3 times/week	1 year	30 minutes	Maintaining a sedentary lifestyle	Baseline ranges: CTX (ng/l): 421 ± 142 (EG), 419 ± 166 (CG) Measurement methods: ECLIA
Baset (24)	Strength Training/High Impact Training	3 times/week	6 mouths	45 minutes	No designated athletic training, just maintaining normal daily activities	Baseline ranges: OC (ng/ml): 13.6 ± 2.4 (SG), 13.6 ± 6.0 (HG), 14.2 ± 3.4 (CG) Measurement method: ELISA
Mosti (25)	Maximum Strength Squat Movement	3 times/week	12 weeks	30–40 minutes	Continue their daily activities and any existing exercise habits	Baseline ranges: CTX (ng/ml): 0.743 ± 0.200 (EG), 0.576 ± 0.051 (CG) P1NP (μg/l): 54.25 ± 15.36 (EG), 51.67 ± 12.36 (CG) Measurement methods: CTX by RIA; P1NP by Serum CrossLaps ELISA
Roghani (26)	Aerobics/Aerobic + Resistance	3 times/week	6 weeks	30 minutes	Daily activities only, no additional exercise interventions	Baseline ranges: P(mg/dl): 3.86 ± 0.40 (AG), 3.33 ± 0.43 (AG+RG), 3.79 ± 0.42 (CG) Ca (mg/dl): 9.10 ± 0.11 (AE), 8.91 ± 0.16 (AG+RG), 9.06 ± 0.38 (CG) ALP (ALP, U/L): 218.00 ± 68.32 (AG), 222.44 ± 60.96 (AG+RG), 181.50 ± 83.36 (CG) Measurement methods: P and Ca: standardized biochemical analysis ALP: ELISA
Pernambuco (27)	Aquatic aerobics exercises	2 times/week	8 months	50 minutes	Daily activities only, no additional exercise interventions	Baseline ranges: OC (ng/ml): 16.4 ± 7.18 (EG), 19.9 ± 5.9 (CG) Measurement method: ELISA
Moreira (28)	High-intensity water exercise	3 times/week	24 weeks	50–60 minutes	Daily activities only, no additional exercise interventions	Baseline ranges: Ca (mg/dL): 9.61 ± 0.33 (EG), 9.50 ± 0.23 (CG) 25(OH)D (nmol/L): 51.8 ± 25.2 (EG), 48.1 ± 19.6 (CG) PTH (pg/mL): 46.62 ± 15.54 (EG), 43.61 ± 15.20 (CG) CTX (ng/mL): 0.330 ± 0.159 (EG), 0.352 ± 0.182 (CG) Measurement method: Ca and 25(OH)D: IRMA PTH: Chemiluminescence immunoassay CTX: ECLIA
Wen (6)	Aerobic exercise	3 times/week	10 weeks	90 minutes	Not participating in any other regular physical activity or sport	Baseline ranges: CTX (nmol/L): 0.69 ± 0.26 (EG), 0.89 ± 0.48 (CG) Measurement method: ELISA
Baker (29)	Whole Body Vibration Training	3 times/week	12 weeks	20 minutes	No designated athletic training, only routine medical care	Baseline ranges: P1NP (μg/L): 62.3 ± 27.0 (EG), 62.2 ± 25.3 (CG) Measurement method: NR
Sen (30)	Whole Body Vibration Training/High Impact Training	3 times/week	24 weeks	20–60 minutes	No designated athletic training, just maintaining normal daily activities	Baseline Ranges: OC (ng/mL): 4.82 ± 1.69 (WG), 2.81 ± 1.84 (HG), 3.66 ± 2.29 (CG) CTX (ng/mL): 0.43 ± 0.23 (WG), 0.45 ± 0.20 (HG), 0.38 ± 0.20 (CG) Measurement methods: OC: Solid phase chemiluminescence CTX: ECLIA
Linero (31)	Resistance exercise	3 times/week	12 weeks	90 minutes	No specific exercise training	Baseline Ranges: CTX (ng/ml): 0.46 ± 0.10 (MHIRT),0.52 ± 0.07 (MHIRT), 0.40 ± 0.05 (LIRT) Measurement method: ECLIA
Pereira (32)	Handball exercise	2–3 times/week	16 weeks	60 minutes	Daily activities only, no additional exercise interventions	Baseline ranges: OC (μg/L): 18.8 ± 7.1 (EG), 16.6 ± 13.1 (CG) P1NP (μg/L): 49.9 ± 18.9 (EG), 38.0 ± 27.5 (CG) Measurement method: Chemiluminescence method (for both OC and P1NP)
Kim (33)	Aerobic + Resistance	2 times/week	6 months	60 minutes	Daily activities only, no additional exercise interventions	Baseline ranges: OC (ng/mL): 8.04 ± 3.93 (EG), 9.18 ± 3.09 (CG) ALP (μg/L): 7.75 ± 4.47 (EG), 10.15 ± 3.91 (CG) Measurement methods: OC: Immunoassay ALP: ELISA
Zaravar (34)	Aquatic aerobics exercises)	3 times/week	8 weeks	60 minutes	Did not train for a specific sport, but maintained their daily activities	Baseline ranges: 25(OH)D (ng/mL): 27.99 ± 6.540 (EG), 28.08 ± 5.995 (CG) PTH (pg/mL): 64.99 ± 14.516 (EG), 64.21 ± 16.097 (CG) Measurement methods: 25(OH)D: ECLIA PTH: Commercial reagent kits
Guzel (35)	Walking	3 times/week	10 weeks	25–40 minutes	Maintain daily activity level without any exercise training	Baseline ranges: 25(OH)D (ng/mL): 18.95 ± 10.08 (EG), 16.62 ± 9.2 (CG) Measurement method: NR
Pasa (36)	Walking and bone joint exercise	3 times/week	8 weeks	30 minutes	Maintain daily habits without any structured physical activity	Baseline ranges: PTH (pg/mL): 151.87 ± 39.84 (Walking), 175.91 ± 57.33 (Bone Joint Exercise), 142.61 ± 26.74 (Control Group, CG) Measurement method: ELISA

EG, exercise group; CG, control group; HG, high intensity group; MG, moderate intensity group; MG, moderate intensity group; WG, whole-body vibration (WBV); SG, strength training group; HIG, high impact training group; AG, aerobic exercise group; RG, resistance exercise group; RIA, radioimmunoassay; MHIRT, moderate to high-intensity resistance training; LIBFR, low-intensity resistance training with blood flow restriction; LIRT, low-intensity resistance training; IRMA, immunoradiometric Assay; ELISA, enzyme-linked immunosorbent Assay; ECLIA, electrochemiluminescence Assay; P, serum phosphorus; Ca, serum calcium; OC, osteocalcin; PTH, parathyroid hormone; ALP, alkaline phosphatase; 25(OH)D, 25-hydroxyvitamin D; CTX, type I collagen cross-linked C-terminal peptide; P1NP, N-terminal propeptide of type I procollagen; NR, not reported.

### 2.5 Risk of bias assessment

For RCTs and clinical controlled trials, the risk of bias was provided by the Review Manager 5.4 self-contained tool (14). Two researchers each assessed the risk of bias in the selected literature using the Cochrane Risk of Bias Assessment Tool. The assessment included randomized sequence generation, allocation concealment, blinding of investigators and subjects, blinded evaluation of study out-comes, completeness of outcome data, selective reporting and other biases. Each factor was assessed as high risk of bias, low risk of bias, or unknown risk of bias. Disagreements that arose during the assessment process were resolved through discussion.

### 2.6 Data analysis

Data were synthesized for the included outcome indicators using Review Manager 5.4 software and stata17 software. The outcome indicators of the studies included in this analysis were all continuous variables. Since all the incorporated studies were RCTs, the between-group differences at baseline should theoretically approach zero. Therefore, post-intervention values were used for the meta-analysis, and standardized mean differences (*SMDs*) with 95% confidence intervals (95% *CIs*) were selected as the effect measures for pooling the effect sizes. Statistical inferences were made through heterogeneity tests and statistical combined effect sizes. The heterogeneity test was evaluated using *I^2^* values. In the heterogeneity test, *P*>0.10 indicated that the heterogeneity of the literature included in this study was negligible, and *P ≤* 0.10 indicated that the heterogeneity of the literature included in this study existed. 0≤*I^2^ ≤* 25% indicated that the heterogeneity was ignored, 25%<*I^2^ ≤* 50% indicated that the inclusion of the literature existed in a mild degree, 50%<*I^2^ ≤* 75% indicated that the inclusion of the study existed in a moderate degree, and *I^2^* >75% indicated that the inclusion of the studies had high heterogeneity. Literature with moderate to high heterogeneity was analyzed using a random effects model, while literature with mild or negligible heterogeneity was analyzed using a fixed effects model.

### 2.7 Subgroup analysis

Subgroup analyses of OC and CTX were conducted according to exercise type, intervention period (≤6 months *vs*. >6 months), and session duration (≤60 minutes *vs*. >60 minutes). Due to the limited number of studies, subgroup analyses were not performed for other bone metabolism markers. In addition, because exercise intensity was inconsistently defined across trials (e.g., based on heart rate, load percentage, or perceived exertion) and only a few studies provided sufficient data, we did not conduct subgroup analyses by intensity.

### 2.8 Sensitivity analysis

We performed sensitivity analysis by eliminating studies one by one to verify the robustness of the results.

### 2.9 Publication bias

We used a funnel plot and Egger’s test to detect publication bias when ≥10 studies with the same outcome were included in the analysis.

### 2.10 Certainty of evidence

We applied the Grading of recommendations assessment, development, and evaluation (GRADE) system to assess the certainty of evidence. Each outcome was evaluated from the following six aspects: study design, risk of bias, inconsistency, indirectness, imprecision and other considerations. Then the certainty of evidence was accordingly graded as “high”, “moderate”, “low”, or “very low” (15). GRADE pro GDT online tool was used to present the summary of findings.

## 3 Results

### 3.1 Results of literature screening

**Figure 1:**
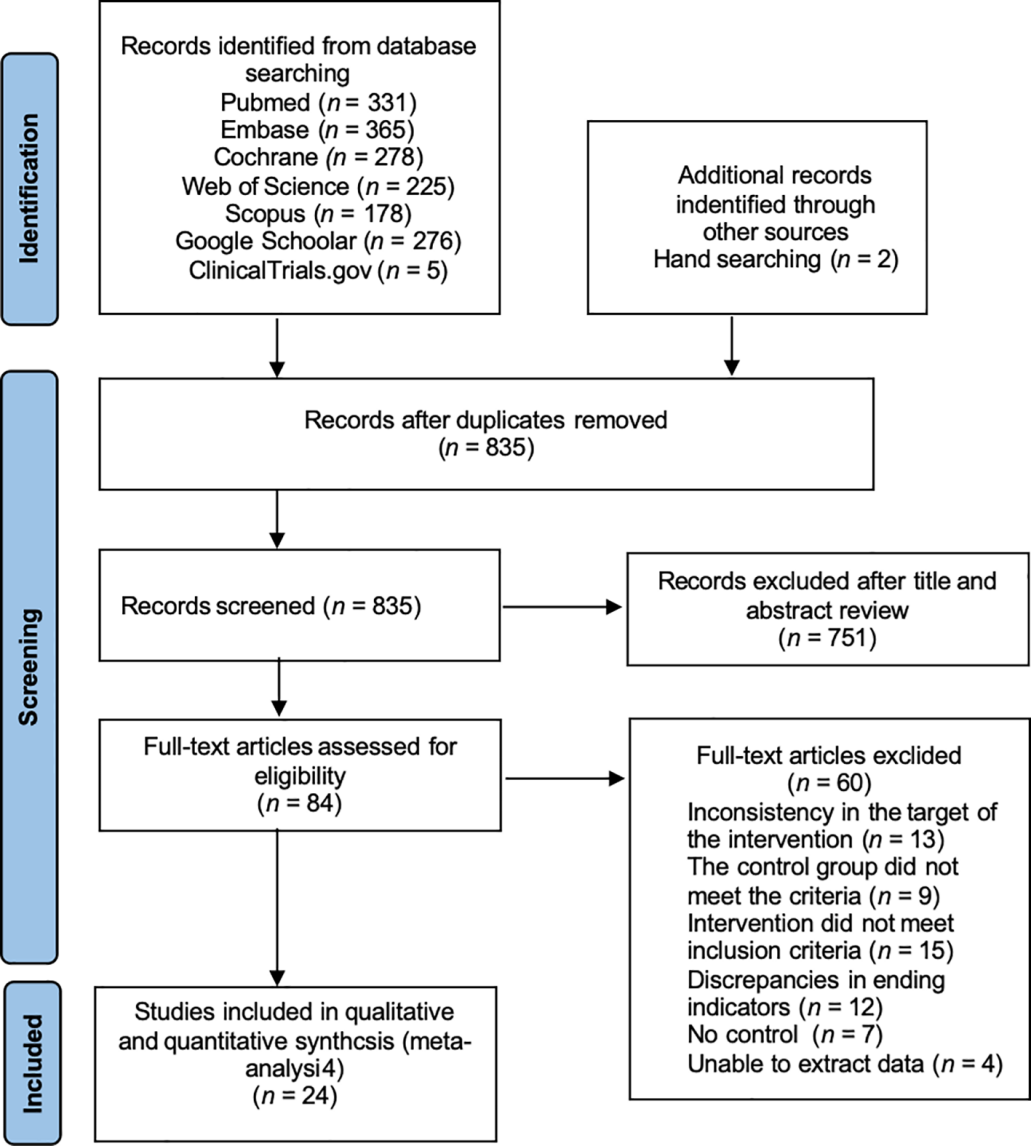
PRISMA study flow diagram.

The initial screening identified 1653 articles, including 331 articles in PubMed database, 365 articles in Embase database, 278 articles in Cochrane Library database, 225 articles in Web of Science database, 178 articles in Scopus database, and 276 articles in Google Scholar database. We obtained 835 articles by de-weighting with EndNote 20 software, excluded 751 articles after reading the titles and abstracts, excluded 7 articles with low relevance, and read through the full text of the remaining 84 articles to assess whether to include them. Among them, 13 interventions did not match the target group, 9 controls did not meet the criteria, 15 interventions did not meet the inclusion criteria, 12 outcome indicators did not match, 7 had no control, and 4 were unable to extract data, and finally, the remaining 24 papers (6, 16–38) were included in the Meta-analysis. (**Figure 1**).

### 3.2 Characteristics of the included studies

A total of 24 papers published between 1993–2024 were included in this study, and the total number of subjects was 1067 (575 in the experimental group and 492 in the control group), aged 45 years or older. Among the countries of publication, five articles were published in Japan (16, 18–20, 28), four in Turkey (21, 24, 30, 35), three in Iran (22, 26, 34), three in the United States (17, 37, 38), one in Brazil (27), one in Sweden (23), one in Norway (25), one in Indonesia (36), Taiwan, China 1 article (6), 1 article (29) in Australia, and 1 article in Portugal (32). The intervention group included 12 articles on aerobic exercise (6, 16, 18–23, 26, 27, 32, 34–36), 4 articles on resistance exercise (17, 24, 25, 31), 3 articles on aerobic combined with resistance exercise (26, 28, 33), 2 articles on whole-body vibration training (29, 30), 2 articles on percussive exercise (24, 30), 2 article on Tai Chi exercise (37, 38). The intervention period was 6 weeks-2 years, the frequency of exercise was 2–4 times/week, and the duration of exercise was 25–90 minutes/session (**Table 2**).

### 3.3 Risk of bias

**Figure 2:**
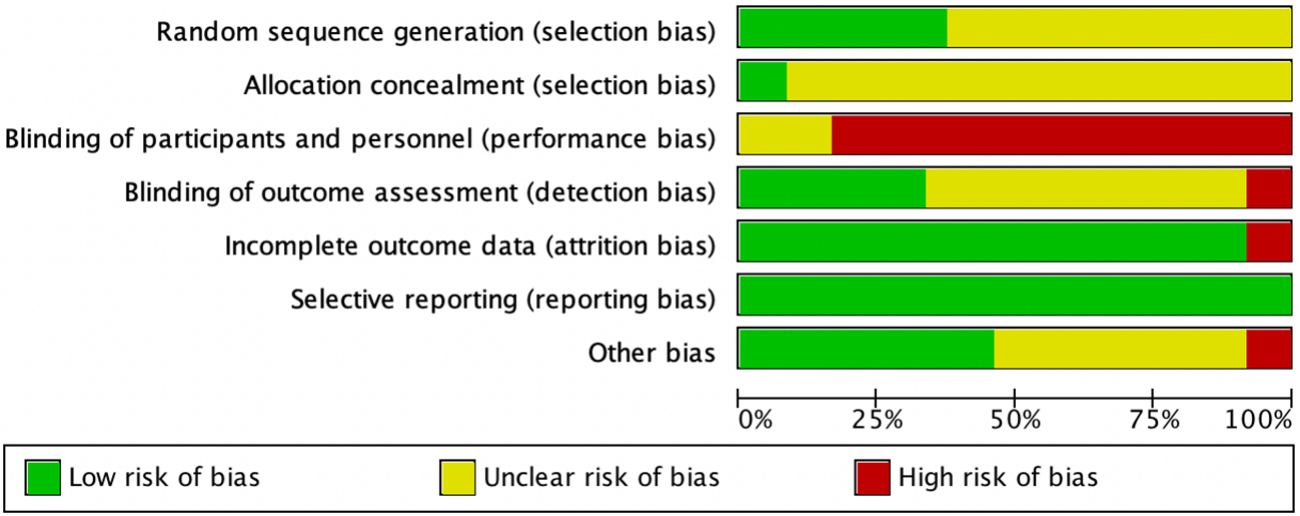
Risk of bias of the included studies.

**Figure 3:**
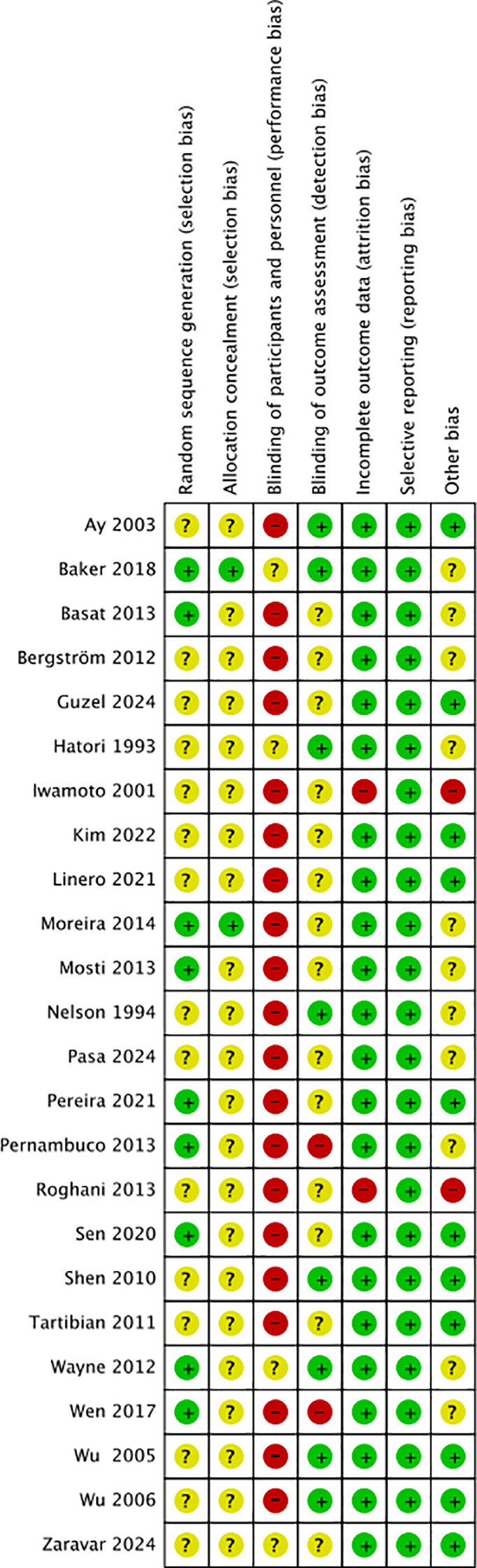
Risk of bias summary of the included studies.

Nine studies (6, 24, 25, 27–30, 32, 37) reported the method of random sequence generation (computer-generated random numbers, variable block randomization, stratified randomization, coin tossing, urn design). One study (29) provided information on allocation concealment, stating that sealed opaque envelopes were used. Twenty-two studies (6, 16–34, 37, 38) reported the number of dropouts and losses to follow-up. Four studies (17, 29, 37, 38) conducted an intention-to-treat (ITT) analysis, while seventeen (6, 16, 18–21, 23–28, 30–33, 37) performed a per-protocol (PP) analysis; four (22, 34–36) did not specify whether ITT or PP was applied. Four studies (29, 30, 37, 38) reported a trial registration number. In summary, since blinding is difficult to implement in exercise intervention studies, most studies were judged to be at “high risk” of bias. The results of the risk of bias assessment are shown in **Figures 2** and **3**.

### 3.4 Meta-analysis results

#### 3.4.1 Effect of exercise on serum phosphorus in postmenopausal women

**Figure 4:**
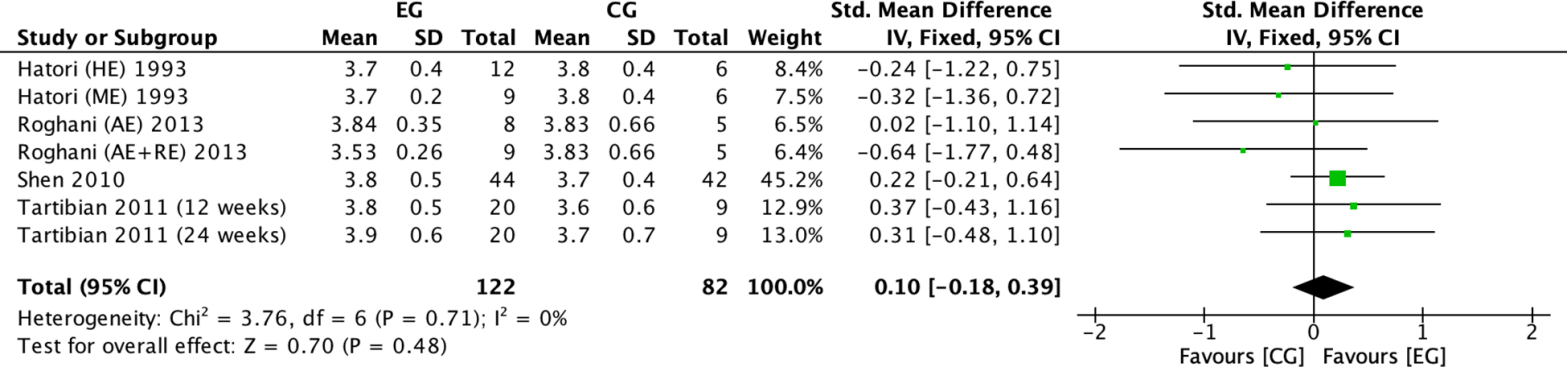
Forest plot of the effect of exercise on serum phosphorus in postmenopausal women. EG, exercise group; CG, control group; HE, high intensity exercise; ME, moderate intensity exercise; AE, aerobic exercise; RE, resistance exercise; SD, standard deviation; Std, standard; IV, inverse variance; df, degrees of freedom.

There were 4 RCTs (16, 18, 22, 38) on the effect of exercise on serum phosphorus in postmenopausal women. Meta-analysis showed negligible heterogeneity of the included studies in the exercise group compared to the control group (*I^2^* = 0%, *P* = 0.71), so a fixed-effects model was used (**Figure 4**). The results showed that serum phosphorus (*SMD* = 0.10, 95% *CI*: -0.18 to 0.39, *P* = 0.48) tended to increase in the exercise group compared to the control group but was not significant. (**Figure 4**).

#### 3.4.2 Effect of exercise on serum calcium in postmenopausal women

**Figure 5:**
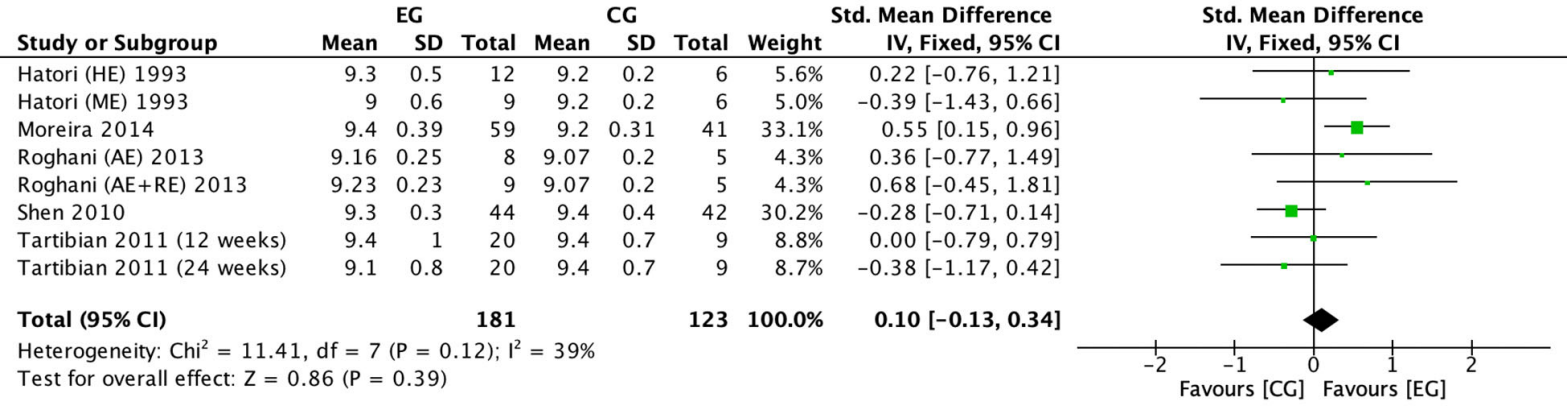
Forest plot of the effect of exercise on serum calcium in postmenopausal women. EG, exercise group; CG, control group; HE, high intensity exercise; ME, moderate intensity exercise; AE, aerobic exercise; RE, resistance exercise; SD, standard deviation; Std, standard; IV, inverse variance; df, degrees of freedom.

There were 5 RCTs (16, 18, 22, 26, 38) on the effect of exercise on serum calcium in postmenopausal women. Meta-analysis showed that the included studies could be mildly heterogeneous in the exercise group compared to the control group (*I^2^* = 39%, *P* = 0.12), so a fixed-effects model was used (**Figure 5**). The results showed that serum calcium (*SMD* = 0.10, 95% *CI*: -0.13 to 0.34, *P* = 0.39) tended to increase in the exercise group compared to the control group but was not significant (**Figure 5**).

#### 3.4.3 Effect of exercise on 25(OH)D in postmenopausal women

**Figure 6:**
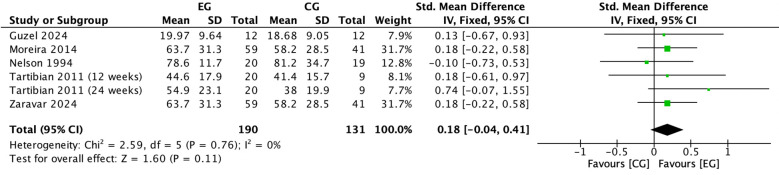
Forest plot of the effect of exercise on 25(OH)D in postmenopausal women. EG, exercise group; CG, control group; SD, standard deviation; Std, standard; IV, inverse variance; df, degrees of freedom.

There were 5 RCTs (17, 22, 28, 34, 35) on the effect of exercise on 25(OH)D in postmenopausal women. Meta-analysis showed negligible heterogeneity in the included studies in the exercise group compared to the control group (*I^2^* = 0%, *P* = 0.76), so a fixed-effects model was used (**Figure 6**). The results showed that serum calcium (*SMD* = 0.18, 95% *CI*: -0.04 to 0.41, *P* = 0.11) tended to increase in the exercise group compared to the control group but was not significant (**Figure 6**).

#### 3.4.4 Effect of exercise on PTH in postmenopausal women

**Figure 7:**
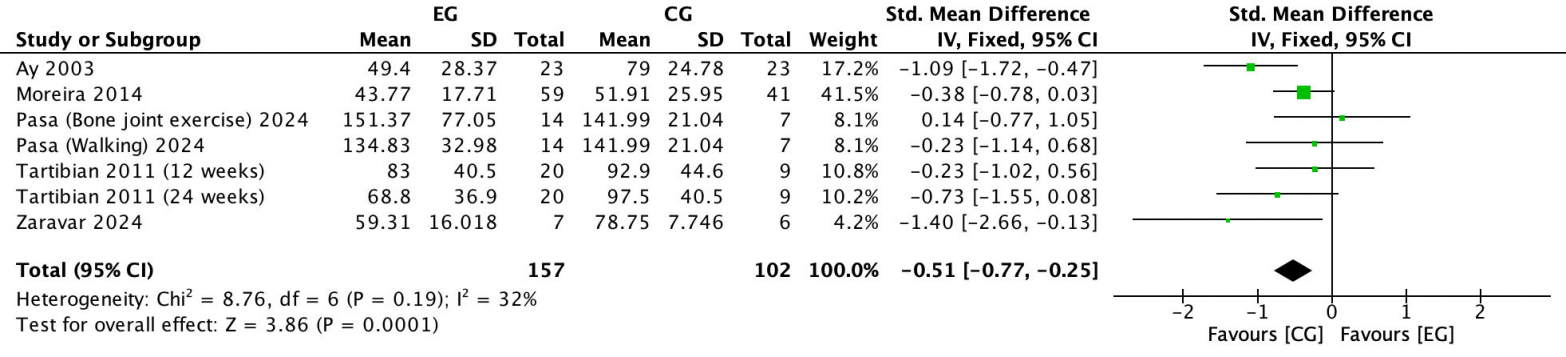
Forest plot of the effect of exercise on PTH in postmenopausal women. EG, exercise group; CG, control group; SD, standard deviation; Std, standard; IV, inverse variance; df, degrees of freedom.

There were 5 RCTs (17, 21, 22, 28, 34) on the effect of exercise on PTH in postmenopausal women. Meta-analysis showed that the included studies could be mildly heterogeneous in the exercise group compared to the control group (*I^2^* = 32%, *P* = 0.19), so a fixed effects model was used (**Figure 7**). The results showed a significant decrease in PTH in the exercise group compared to the control group (*SMD*=-0.51, 95% *CI*: -0.77 to -0.25, *P* = 0.0001) (**Figure 7**).

#### 3.4.5 Effect of exercise on ALP in postmenopausal women

**Figure 8:**
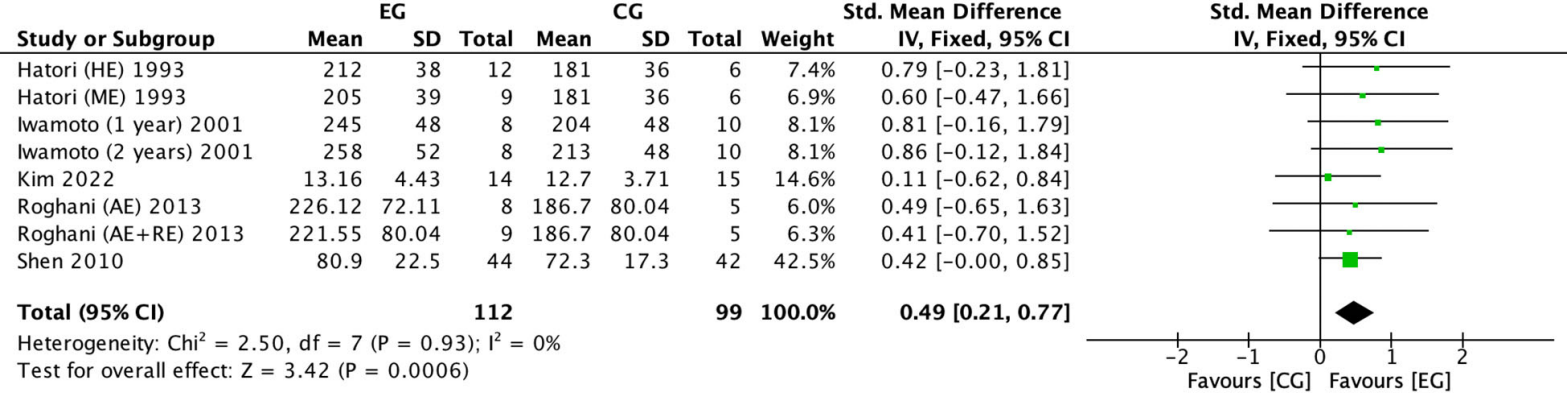
Forest plot of the effect of exercise on ALP in postmenopausal women. EG, exercise group; CG, control group; HE, high intensity exercise; ME, moderate intensity exercise; AE, aerobic exercise; RE, resistance exercise; SD, standard deviation; Std, standard; IV, inverse variance; df, degrees of freedom.

There were 5 RCTs (16, 18, 26, 33, 38) on the effect of exercise on ALP in postmenopausal women. Meta-analysis showed negligible heterogeneity in the included studies in the exercise group compared to the control group (*I^2^* = 0%, *P* = 0.93), so a fixed-effects model was used (**Figure 8**). The results showed a significant increase in ALP in the exercise group compared to the control group (*SMD* = 0.49, 95%*CI*: 0.21 to 0.77, *P* = 0.0006) (**Figure 8**).

#### 3.4.6 Effect of exercise on P1NP in postmenopausal women

**Figure 9:**
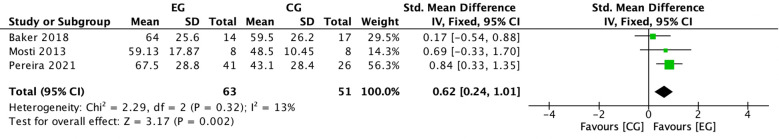
Forest plot of the effect of exercise on P1NP in postmenopausal women. EG, exercise group; CG, control group; SD, standard deviation; Std, standard; IV, inverse variance; df, degrees of freedom.

There were 3 RCTs (25, 29, 32) on the effect of exercise on P1NP in postmenopausal women. Meta-analysis showed negligible heterogeneity in the included studies in the exercise group compared to the control group (*I^2^* = 13%, *P* = 0.32), so a fixed-effects model was used (**Figure 9**). The results showed a significant increase in P1NP in the exercise group compared to the control group (*SMD* = 0.62, 95% *CI*: 0.24 to 1.01, *P* = 0.002) (**Figure 9**).

#### 3.4.7 Effect of exercise on CTX in postmenopausal women

**Figure 10:**
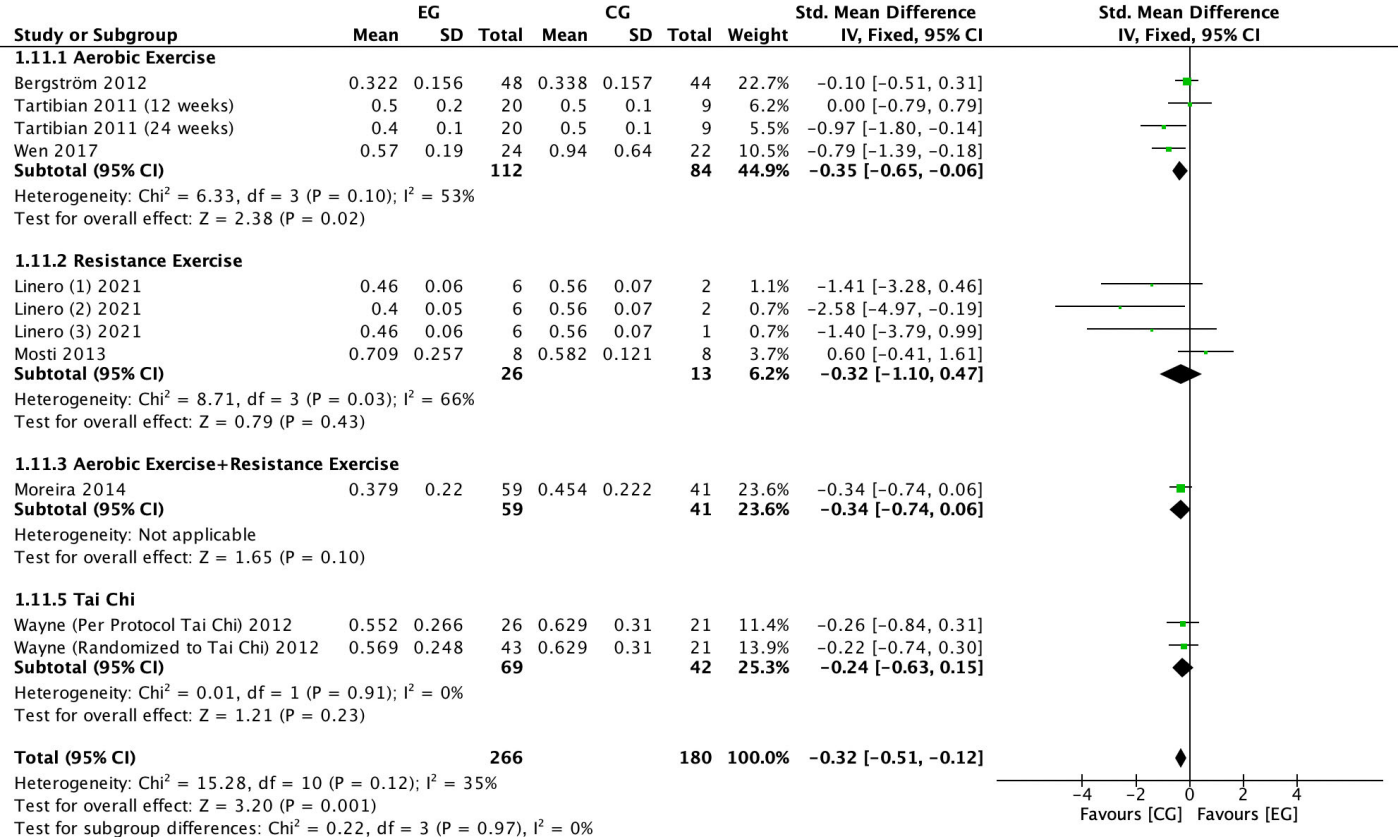
Forest plot of the effect of exercise on CTX in postmenopausal women (subgroup analysis of exercise type). EG, exercise group; CG, control group; SD, standard deviation; Std, standard; IV, inverse variance; df, degrees of freedom.

**Figure 11:**
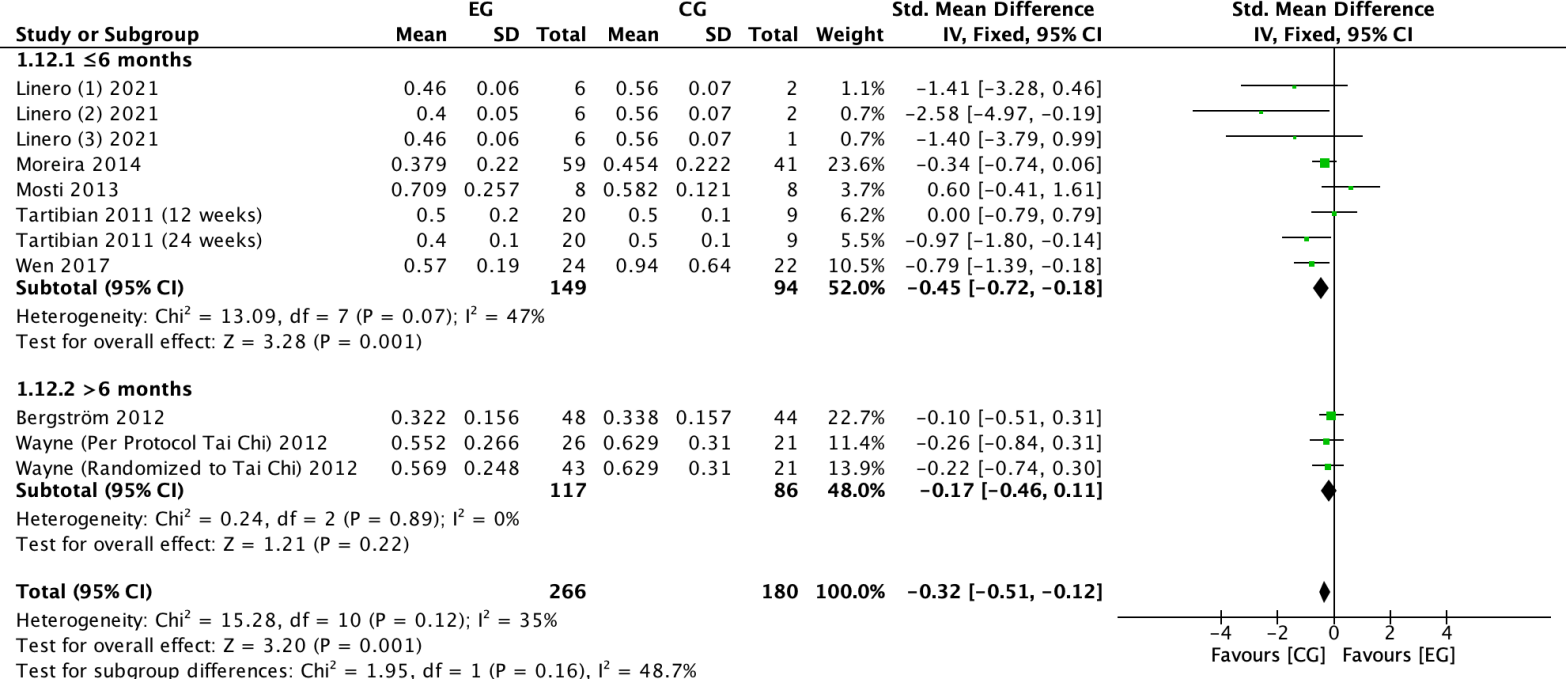
Forest plot of the effect of exercise on CTX in postmenopausal women (subgroup analysis of exercise cycles). EG, exercise group; CG, control group; SD, standard deviation; Std, standard; IV, inverse variance; df, degrees of freedom.

**Figure 12:**
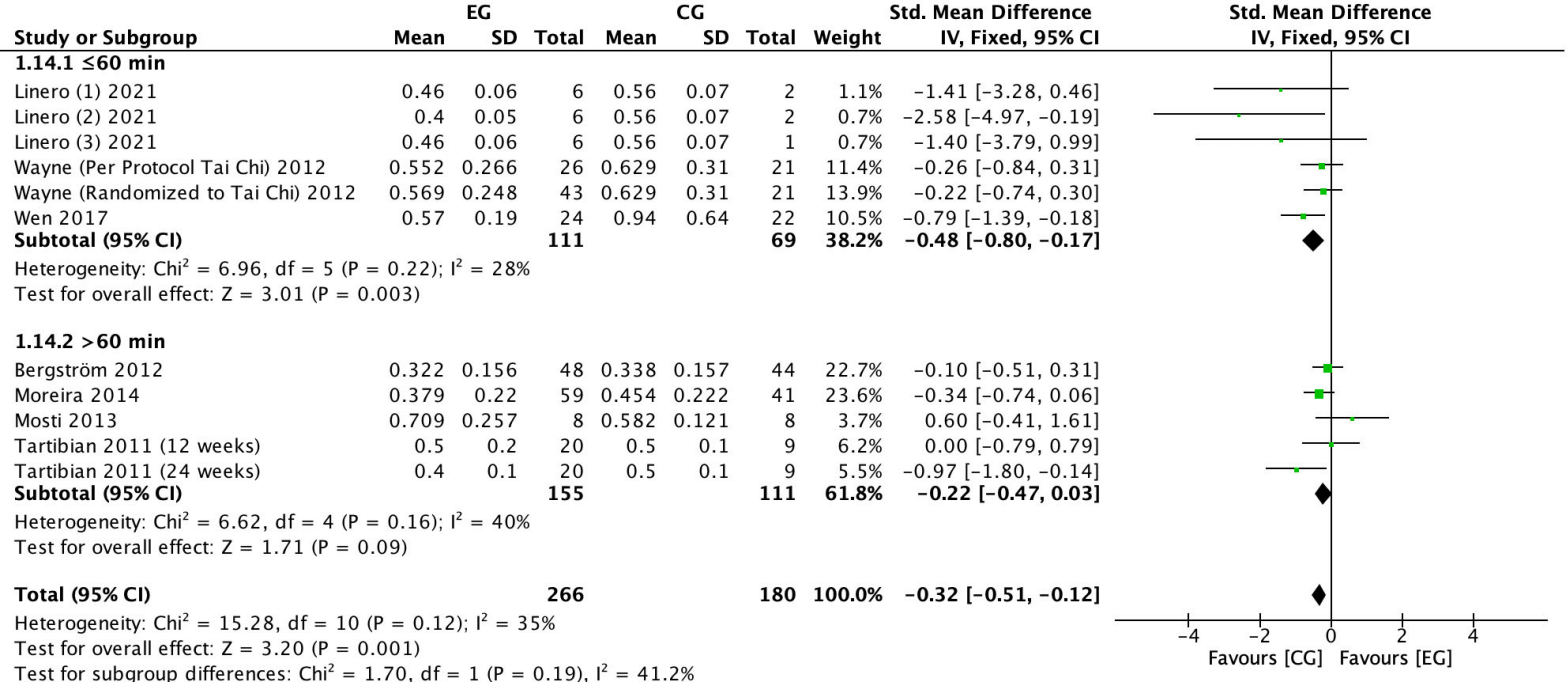
Forest plot of the effect of exercise on CTX in postmenopausal women (subgroup analysis of the duration of a single exercise session). EG, exercise group; CG, control group; SD, standard deviation; Std, standard; IV, inverse variance; df, degrees of freedom.

There were 7 RCTs (6, 22, 23, 25, 28, 31) on the effect of exercise on CTX in postmenopausal women. Meta-analysis showed moderate heterogeneity of the included studies in the exercise group compared to the control group (*I^2^* = 35%, *P* = 0.12), so a fixed-effects model was used (**Figures 10**-**12**). The results showed that the CTX levels in the exercise group were significantly lower than those in the control group (*SMD* = -0.32, 95% *CI*: -0.51 to -0.12, *P* = 0.001). The results of the subgroup analysis by exercise type showed that aerobic exercise (*SMD* = -0.35, 95% *CI*: -0.65 to -0.06, *P* = 0.02) significantly reduced CTX levels; resistance exercise (*SMD* = -0.32, 95% *CI*: -1.10 to 0.47, *P* = 0.43), combined aerobic plus resistance exercise (*SMD* = -0.34, 95% *CI*: -0.74 to 0.06, *P* = 0.10) and Tai Chi (*SMD* = -0.24, 95% *CI*: -0.63 to 0.15, *P* = 0.23) showed a trend toward reducing CTX, but without statistical significance (**Figure 10**). The further stratification of sample size may have resulted in smaller pooled effect sizes and an insufficient number of studies, leading to unstable statistical results and less convincing findings. The results of cycle subgroup analysis showed that exercise for ≤6 months (*SMD*=-0.45, 95% *CI*: -0.72 to -0.18, *P* = 0.001) significantly reduced CTX levels; exercise for >6 months (*SMD*=-0.17, 95% *CI*: 0.46 to 0.11, *P* = 0.22) had a trend to reduce CTX levels and was not significant (**Figure 11**). Subgroup analysis of the duration of a single exercise session showed that exercise of ≤60 min (*SMD*=-0.48, 95% *CI*: -0.80 to -0.17, *P* = 0.003) significantly reduced CTX levels, and exercise of >60 min (*SMD*=-0.22, 95% *CI*: -0.47 to 0.03, *P* = 0.09) had a trend to reduce CTX levels and was not significant (**Figure 12**).

#### 3.4.8 Effect of exercise on OC in postmenopausal women

**Figure 13:**
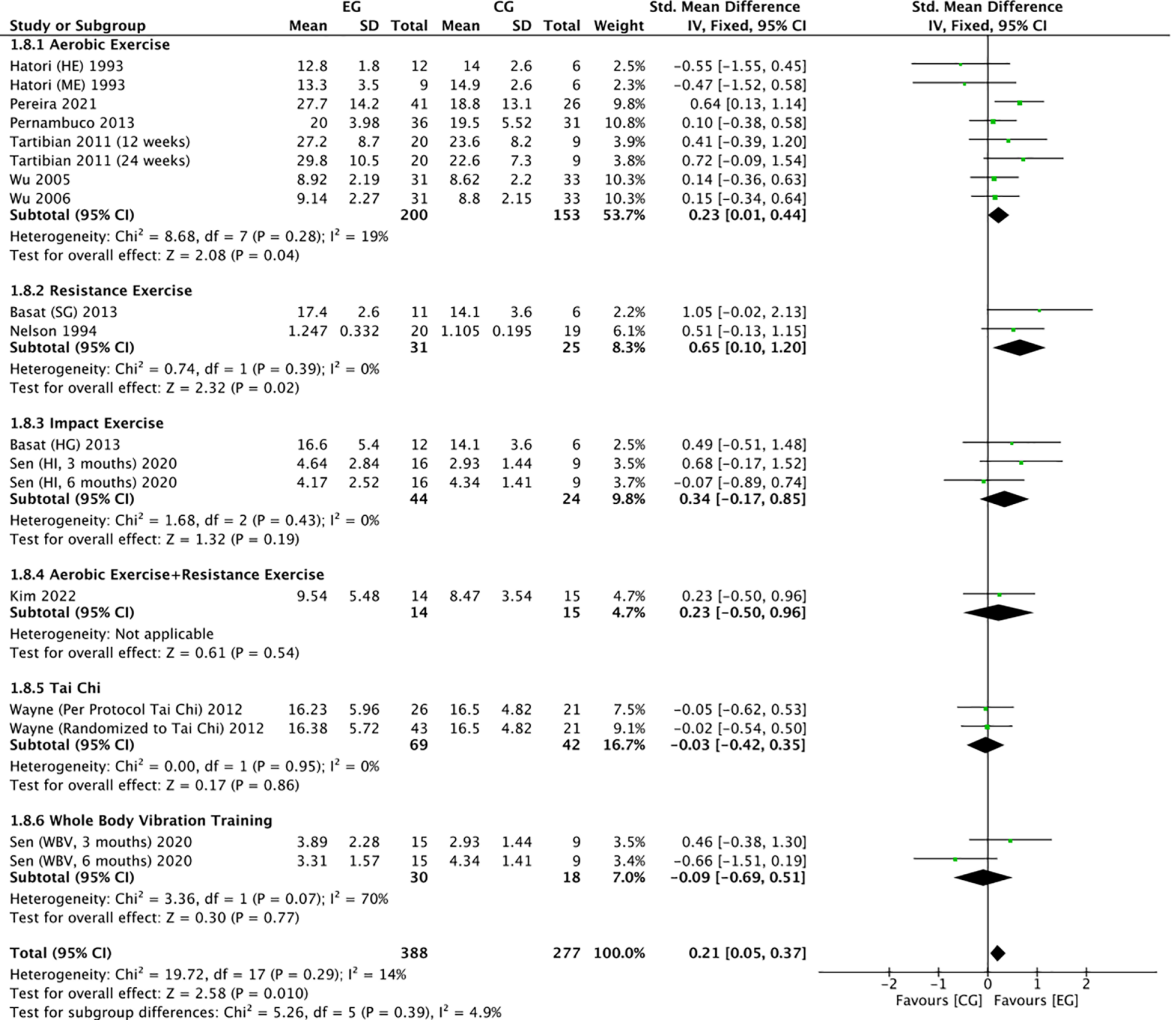
Forest plot of the effect of exercise on OC in postmenopausal women (exercise type subgroup analysis). EG, exercise group; CG, control group; HE, high intensity exercise; ME, moderate intensity exercise; SG, strength training group; HG, high impact training group; WBV, whole body vibration; HI, high impact; SD, standard deviation; Std, standard; IV, inverse variance; df, degrees of freedom.

**Figure 14:**
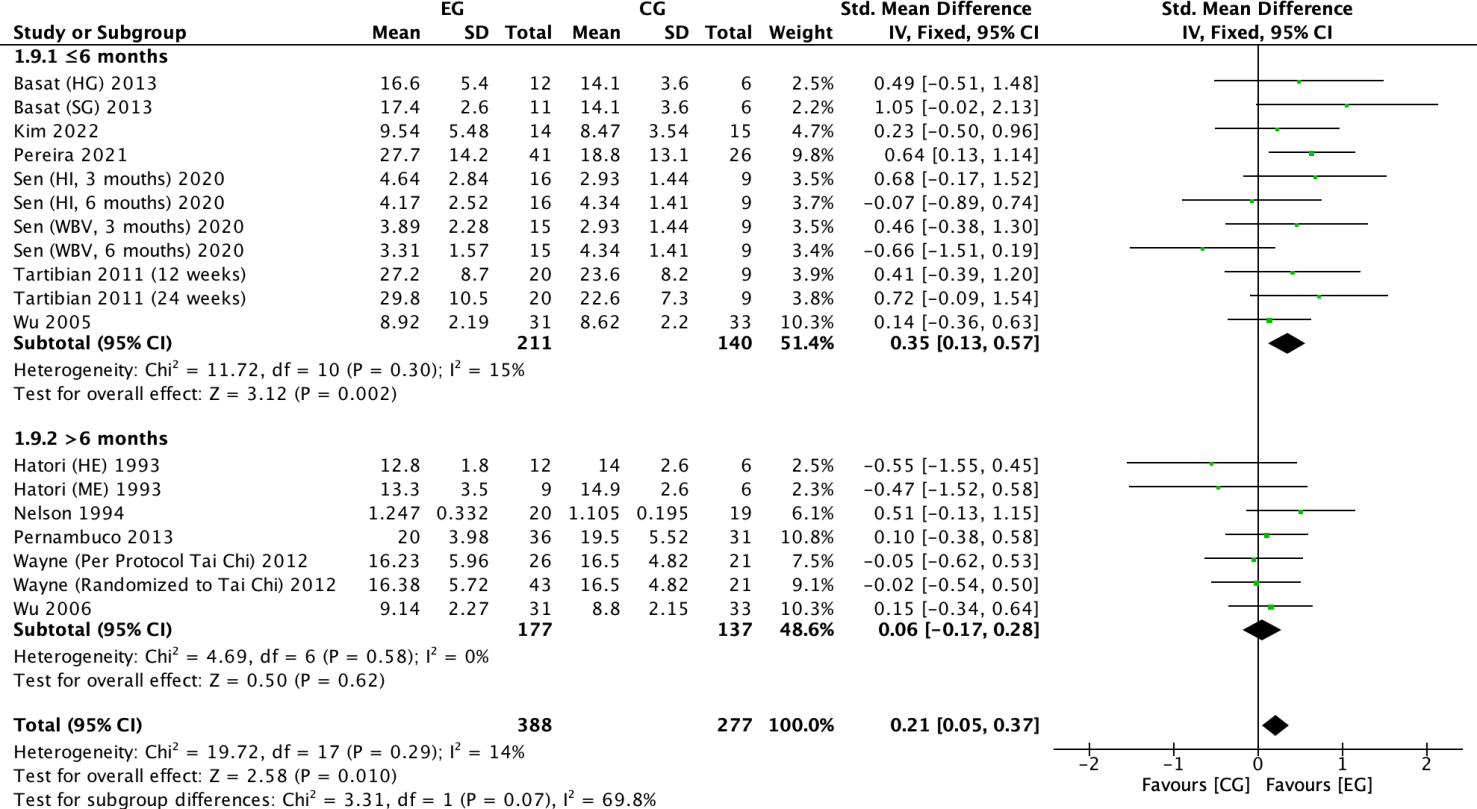
Forest plot of the effect of exercise on OC in postmenopausal women (exercise cycle subgroup analysis). EG, exercise group; CG, control group; HE, high intensity exercise; ME, moderate intensity exercise; SG, strength training group; HG, high impact training group; WBV, whole body vibration; HI, high impact; SD, standard deviation; Std, standard; IV, inverse variance; df, degrees of freedom.

**Figure 15:**
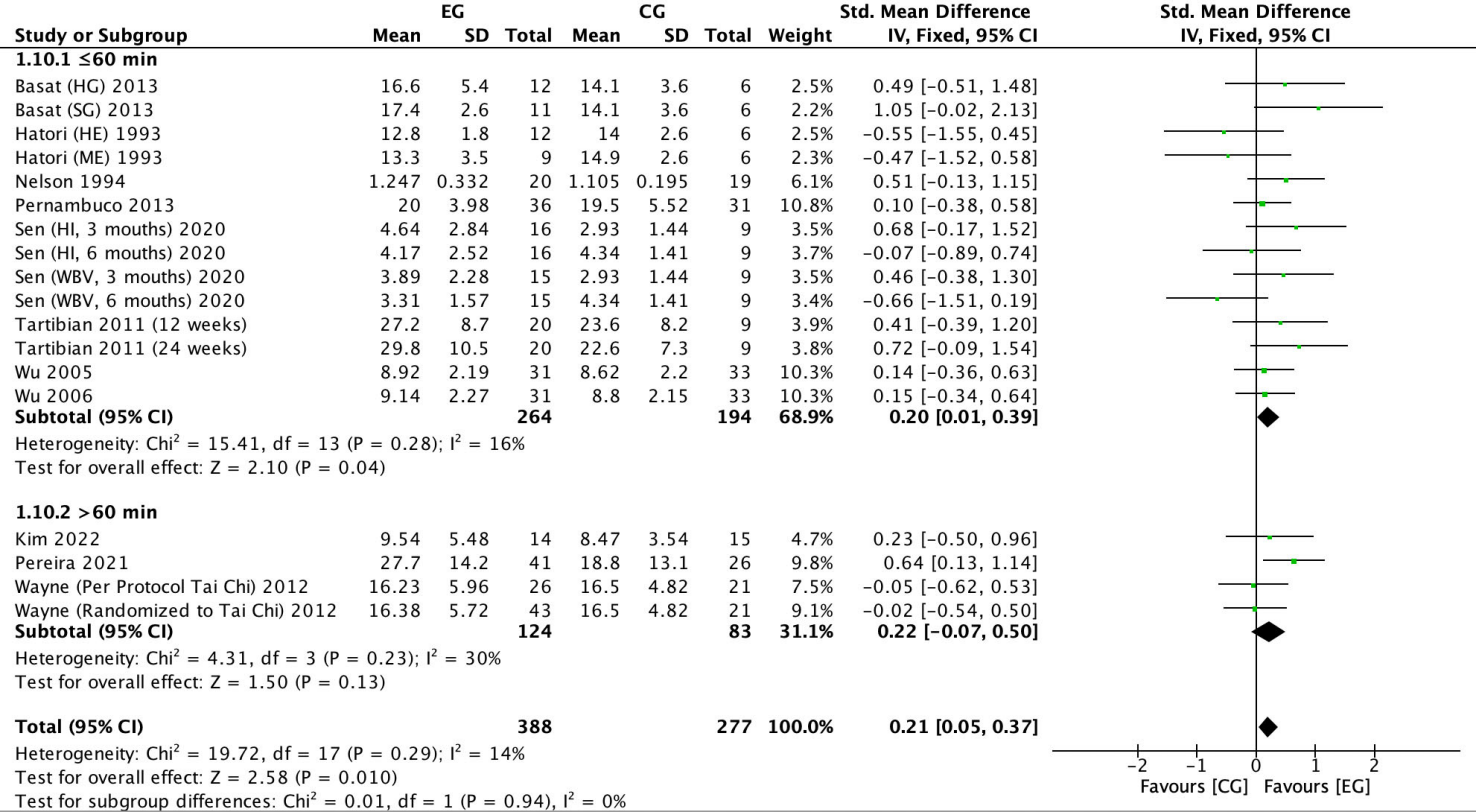
Forest plot of the effect of exercise on OC in postmenopausal women (subgroup analysis of the duration of a single exercise session). EG, exercise group; CG, control group; HE, high intensity exercise; ME, moderate intensity exercise; SG, strength training group; HG, high impact training group; WBV, whole body vibration; HI, high impact; SD, standard deviation; Std, standard; IV, inverse variance; df, degrees of freedom.

There were 11 RCTs (16, 17, 19, 20, 22, 23, 25, 27, 32, 33, 37)on the effect of exercise on OC in postmenopausal women. Meta-analysis showed moderate heterogeneity of the included studies in the exercise group compared to the control group (*I^2^* = 14%, *P* = 0.29), so a fixed-effects model was used (**Figures 13**-**15**). The results showed a trend of increasing OC in the exercise group compared to the control group (*SMD* = 0.21, 95% *CI*: 0.05 to 0.37, *P* = 0.01) and were not significant. The subgroup analysis by exercise type showed that aerobic exercise (*SMD* = 0.23, 95% *CI*: 0.01 to 0.44, *P* = 0.04) and resistance exercise significantly (*SMD* = 0.65, 95% *CI*: 0.10 to 1.20, *P* = 0.02) increased OC levels; impact exercise (*SMD* = 0.34, 95% *CI*: -0.17 to 0.85, *P* = 0.19), combined aerobic plus resistance exercise (*SMD* = 0.23, 95% *CI*: -0.50 to 0.96, *P* = 0.54), Tai Chi (*SMD* = -0.03, 95% *CI*: -0.42 to 0.35, *P* = 0.86) and whole body vibration training (*SMD* = -0.09, 95% *CI*: -0.69 to 0.51, *P* = 0.77) showed a trend toward reducing OC, but without statistical significance (**Figure 13**). The refinement of sample size classification may have resulted in a smaller pooled effect size, and the insufficient number of studies led to unstable statistical results, making the findings less convincing. Cycle time subgroup analysis showed that exercise ≤6 months (*SMD* = 0.35, 95% *CI*: 0.13 to 0.57, *P* = 0.002) significantly elevated OC. exercise >6 months (*SMD* = 0.06, 95% *CI*: -0.17 to 0.28, *P* = 0.62) showed a trend toward increasing OC levels and was not significant (**Figure 14**). Subgroup analysis of the duration of a single exercise session showed that exercise for ≤60 min (*SMD* = 0.20, 95% *CI*: 0.01 to 0.39, *P* = 0.04) elevated OC content; exercise for >60 min (*SMD*=-0.22, 95% *CI*: -0.07, 0.50, *P* = 0.13) tended to decrease OC content and was non-significant (**Figure 15**). 11 studies reported the effect of exercise on OC in postmenopausal women, so we assessed their publication bias. The funnel plot (**Supplementary Figure S1**) and Egger’s test (*P* = 0.953) (**Table 3**) showed no evidence of publication bias.

**Table 3:** Egger test results.

Std_Eff	Coefficient	Std. err	*t*	*P*>*|t|*	(95% conf. interval)
slope	.1926973	.3412466	0.56	0.580	-.5307131.9161077
bias	.0602132	.9994193	0.06	0.953	-2.058461 2.178887

### 3.5 Sensitivity analysis

The effect sizes after each indicator were removed from the study on a study-by-study basis were within the 95%CI of the total effect size, and therefore the effect on the total combined effect size was small and acceptable, strengthening the results of the original meta-analysis and making it more convincing (**Supplementary Figures S2-S9**).

### 3.6 Certainty of evidence

The certainty of evidence was rated as low for two outcomes (CTX and OC). The evidence of the remaining outcomes was rated as very low certainty. The reasons for downgrading were mainly attributed to the risk of bias of included studies and imprecision. The results of certainty of evidence are shown in **Supplementary Table S3**.

## 4 Discussion

Exercise, as an effective non-pharmacological intervention, may be the main reason for the improvement of bone health since bone responds to mechanical loads and acts on the skeleton through muscular forces and ground reaction forces, which increase the density and strength of bone minerals (39). Exercise is widely recommended for the prevention of osteoporosis due to the osteogenic effects of these forces and the lack of side effects (39). To investigate the role of exercise on bone metabolism in postmenopausal women, the present study conducted a meta-analysis of relevant RCTs published to date, which is the gold standard for evaluating interventions and resides at the top of the evidence hierarchy for individual studies. The overall quality of the 24 RCTs included in the study was high, thus enhancing the reliability of the findings.

The results of the subgroup analysis in this study indicate that an exercise duration of ≤6 months and a session length of ≤60 minutes are more effective in reducing the bone resorption marker CTX, while significantly increasing the bone formation marker OC. Exercise exerts mechanical loading on bone tissue, which suppresses the secretion of sclerostin by osteocytes, thereby relieving the inhibition of the Wnt/β-catenin signaling pathway. This promotes the intracellular accumulation of β-catenin and its translocation into the nucleus, where it activates the expression of bone formation–related genes and enhances osteogenic activity (40–42). This pathway not only facilitates the differentiation and proliferation of osteoblasts but also upregulates osteoprotegerin expression, further inhibiting osteoclast activity and collectively shifting bone metabolism toward a state that favors bone formation (42, 43). In addition, Bone responds to mechanical loading with rapid saturation and recovery of sensitivity: during a single training bout, a limited number of loading cycles is sufficient to trigger osteogenic signaling, and prolonging the duration or increasing the number of cycles does not proportionally enhance the bone-forming effect (44). Short-duration (≤60 min) and regular mechanical loading, combined with adequate rest intervals, can effectively maintain the mechanosensitive of bone cells and thereby enhance bone metabolic responses (45). In addition, bone turnover markers such as P1NP and CTX have been shown to change significantly within several weeks to months of exercise intervention, suggesting that an intervention period of ≤6 months is sufficient to induce measurable improvements in bone metabolism (46). When mechanical loading is maintained in a fixed pattern over a long term, the mechanosensitive of bone cells gradually declines, leading to a “desensitization” phenomenon and reduced bone formation efficiency per training unit (47). Furthermore, long-term interventions are more likely to be affected by reduced adherence, soft tissue fatigue, hormonal fluctuations, and limited energy availability, all of which can attenuate positive adaptations in bone metabolism (48). Therefore, exercise prescriptions should emphasize moderate session duration, regular frequency, and periodic adjustments, rather than simply extending the intervention time, to avoid plateaus and maximize the benefits of bone remodeling.

In their meta-analysis on the effects of Tai Chi on bone health in postmenopausal women, Liu et al. selected “6 months” as the cutoff point for intervention duration based on the physiological rationale of the bone remodeling cycle (49). The bone remodeling cycle typically takes 3–4 months to complete the sequential processes of bone resorption, formation, and mineralization, while achieving a new steady-state bone mass requires at least 6–8 months (50). Therefore, a 6-month intervention period ensures coverage of a full bone metabolic cycle, allowing for a more accurate assessment of the cumulative effects of exercise on bone mineral density.

Most meta-analyses related to the improvement of bone health by exercise have focused on bone mineral density (9, 51–54). In contrast, there are relatively few in-depth studies on the effects of exercise on bone metabolism. The health of bone metabolism is crucial for maintaining bone strength and stability, which involves multiple aspects of bone growth, remodeling, and repair. By promoting bone metabolism, it not only enhances the stress resistance of bones and reduces the risk of fracture, but also improves the bone microstructure and enhances the overall quality of bones. Therefore, strengthening research on the relationship between exercise and bone metabolism is of great significance in preventing osteoporosis and promoting bone health. Under physiological conditions, bone resorption and bone formation maintain a dynamic balance in bone metabolism (55). Bone metabolism markers are metabolites produced in the process of bone transformation and play an important role in the diagnosis and treatment of osteoporosis because they can timely and accurately reflect the state of bone transformation in the human body (56). Bone metabolism includes two processes: bone resorption and bone formation. Bone resorption is a process in which osteoclasts break down part of the bone and form a cavity (bone resorption cavity), during which bone metabolites are formed that can enter the bloodstream or be excreted through urine, a process that lasts 4–6 weeks (57). Bone formation, on the other hand, takes place in the cavity formed by bone resorption, during which molecules secreted by osteoblasts can enter the bloodstream while completing cavity ossification (58). Bone formation markers reflect the activity of osteoblasts, and the bone formation markers included in this exploration are OC, ALP, BALP, and P1NP.Bone resorption markers reflect the activity of osteoclasts, and the bone resorption markers included in this study are CT. It has been suggested that bone resorption may have an impact on bone health in the context of reduced or unchanged bone resorption (59). In terms of predicting fracture risk, bone metabolism markers have been correlated with fracture risk, with higher concentrations of bone metabolism markers being associated with greater fracture risk (12). Other studies have shown that bone metabolism markers are more useful in understanding the state of bone mass by their ability to capture information about bone transition state, which is the 2nd most important factor in the development of osteoporosis and the occurrence of fragility fractures, as opposed to BMD by measuring bone mass (60). It has also been suggested that bone metabolic markers may influence fracture risk independently of BMD (by influencing bone strength) (61). Bone metabolism markers have a unique advantage in monitoring therapy in that they respond rapidly to changes in bone physiology and remain relatively stable, which can help physicians to have sufficient response time to determine adjustments to treatment regimens in the event of poor efficacy, which can be particularly useful in patients with demanding treatment regimens such as bisphosphonate therapy (62). In conclusion, through the unremitting efforts of researchers, bone metabolism markers are becoming richer and richer, their application aspects are becoming broader and broader, and their role in the diagnosis and treatment of osteoporosis is becoming more and more prominent, but more research is needed to improve the diagnostic and therapeutic system of bone metabolism biochemical markers to be widely used in the clinic.

The results of the meta-analysis showed that, compared with the control group, exercise was effective in increasing the levels of bone formation markers, such as ALP, P1NP, and OC, in postmenopausal women. ALP on the other hand widely present in various tissues of the human body, serum ALP is mainly derived from liver, bone and kidney tissues, it is a marker of bone formation, and studies suggest that ALP has an important role in the pathogenesis of osteoporosis (63). Serum ALP levels are significantly elevated in pathological conditions, such as the occurrence of diseases of the hepatobiliary system, as well as bone diseases (64). The level of P1NP in the serum reflects the ability of osteoblasts to synthesize osteoclasts collagen and forms the basis of a laboratory index for monitoring osteoblast viability and bone formation (65). Its blood level mainly reflects the rate of type I collagen synthesis and bone conversion, and is a specific and sensitive indicator of new bone formation (66).The results of Kohrt et al. (67) showed that P1NP increased with exercise (P<0.001), suggesting that exercise promotes the synthesis of P1NP, which, in turn, enhances osteoclast viability and promotes bone formation. OC is a protein secreted by osteoblasts secreted protein that plays an important role in maintaining bone health (68). Numerous studies have shown that exercise can promote osteogenic differentiation of bone marrow mesenchymal stem cells and osteoblasts, promote bone formation, improve bone metabolism, and thus prevent and control osteoporosis (69).OC is a key osteogenic factor in the process of bone formation, and thus exercise can promote blood circulation and metabolism in the skeleton, which may stimulate osteoblasts to secrete more OC (70). Exercise activates the mechanotransduction pathways in osteocytes by applying mechanical load, thereby upregulating the expression of osteogenic markers such as OC and ALP, while simultaneously inhibiting bone resorption by regulating the balance of osteoprotegerin (OPG) and receptor activator of nuclear factor κB ligand (RANKL). Specifically, exercise increases OPG expression and decreases RANKL expression, thereby suppressing osteoclast differentiation and activity (71). Animal studies have shown that treadmill and vibration training can reduce RANKL and increase OPG (72), and mechanical strain can directly inhibit RANKL expression (73).

The results of this study showed that exercise significantly reduced the levels of bone resorption markers such as PTH and CTX in postmenopausal women. PTH is a hormone that regulates calcium and phosphorus metabolism, and 15.6% of postmenopausal osteoporosis patients had elevated PTH. The results of the study on the correlation between osteoporosis-related hormones and bone mineral density showed that the level of serum PTH was negatively correlated with bone mineral density, which allowed early detection of osteoporosis (74). Its reduction by exercise may imply improve bone health in middle-aged and older adults. CTX is the most widely used marker of collagen degradation, and the level of CTX reflects the bone resorption activity of osteoclasts. CTX is a valid marker for metabolic bone diseases characterized by significantly increased osteoclast activity (75). CTX correlates with the degree of bone resorption and responds rapidly and sensitively to antiresorptive therapy. Detection of serum CTX levels can predict the severity of bone conversion, and serve as a clinically important reference index for assessing bone conversion-related diseases (58). Exercise-induced changes in PTH and CTX concentration levels are consistent with a study on the effects of bath therapy and aquatic exercise on these two hormones (11).

The results also showed that exercise did not significantly affect serum phosphorus, serum calcium and 25(OH)D in postmenopausal women. Serum phosphorus and serum calcium play several important roles in bone metabolism with exercise intervention, which is involved in bone formation and repair, influences changes in bone metabolism markers, regulates acid-base balance and cellular osmotic pressure, and interacts with calcium and phosphorus (76–78). For serum phosphorus and serum calcium, exercise may indeed have some effect on them, but the exact effect varies from person to person. Exercise promotes an increase in metabolic rate, which in turn affects the phosphorus-calcium balance in the blood (79). 25(OH)D is the main form of vitamin D in the body and is essential for calcium absorption and bone health (80). 25(OH)D acts as a calcium-regulating hormone that inhibits the elevation of PTH, increases osteomineralization to prevent bone loss, strengthens muscles, improves balance, and prevents falls in the elderly (80). Groenendijk (81) and others showed that fortified milk supplementation and exercise intervention successfully improved 25(OH)D concentrations and the balance of bone turnover markers in Chinese middle-aged and elderly people. The effects of exercise on bone metabolism are complex, and although serum phosphorus, serum calcium, and 25(OH)D play important roles in bone health, the specific effects of exercise on serum phosphorus, serum calcium, and 25(OH)D may not be significant, possibly due to differences in exercise modalities and intensities, as well as individual differences.

Our subgroup analysis demonstrated that both aerobic and resistance exercise significantly increased OC levels in postmenopausal women, while aerobic exercise also reduced CTX levels. Previous studies suggest that aerobic exercise may increase osteoprotegerin levels, which helps suppress osteoclastogenesis and reduce bone loss, thereby leading to decreased serum CTX and elevated OC levels (23, 82). In contrast, resistance exercise promotes osteoblast activity through direct mechanical loading, resulting in increased OC levels (82, 83). High-load and explosive training methods enhance one-repetition maximum (1RM) strength and rate of force development, thereby stimulating bone formation, increasing bone mineral content, and potentially triggering adaptive skeletal responses through mechanical stress (25). High-impact exercise, by increasing mechanical loading on the skeleton, significantly improves bone mineral density of the lumbar spine and femoral neck, while also promoting an increase in bone formation markers (e.g., OC) and reducing bone resorption markers (e.g., P1NP), thus improving bone metabolism in postmenopausal women with osteoporosis (30). Tai Chi, as a mind–body exercise, may indirectly reduce fall risk by improving balance and muscle strength, while also slowing bone loss and slightly increasing bone density (e.g., in the femoral neck region) through moderate mechanical loading (37). Whole-body vibration training promotes bone formation and reduces bone resorption through high-frequency, low-amplitude mechanical stimulation, thereby increasing bone density and mechanical strength, with particularly notable effects in the femoral neck and lumbar spine regions (29).

Limitations: (i) As the study was an analysis of different bone metabolism by different exercise types, the refinement of the sample size classification may have resulted in a smaller amount of combined effects, which may have had a certain impact on the results, so the analysis was not carried out on the exercise types; (ii) The included studies exhibited heterogeneity in the methods used to measure bone metabolism markers (e.g., IRMA, ELISA), which may affect the comparability and synthesis of the results; (iii) Most of the included studies involved short-term interventions and lacked long-term follow-up data (>2 years); therefore, the sustainability of the intervention effects and their long-term impact on bone health could not be clearly evaluated; (iv) Since all included studies were RCTs, the between-group differences at baseline should theoretically approach zero, which justified our use of post-intervention values for the meta-analysis. However, although randomization balances baseline differences, potential confounding factors may not be fully controlled, which could introduce some bias into the results. Future studies should prioritize reporting baseline data and change-from-baseline values to allow for more comprehensive analyses; (v) This study only searched English-language databases, potentially omitting relevant studies published in other languages (e.g., Chinese, Spanish), which may limit the comprehensiveness and representativeness of the results. Future research should include multilingual databases to reduce potential language bias; (vi) The applicability of our study results to populations from different regions or ethnic backgrounds may be limited, particularly in groups with substantial differences in baseline bone health, lifestyle, or genetic background, and thus should be generalized with caution; (vii) In the study, although the 6-month intervention duration was justified based on the physiological rationale of the bone remodeling cycle, the cut-off point of 60 minutes for single-session exercise duration lacks a clear physiological basis. The choice of this cut-off may be more related to the distribution of the data or operational convenience rather than physiological significance. Therefore, we acknowledge the potential bias that may be introduced by this arbitrary cut-off.

## 5 Conclusion

The systematic review and meta-analysis of this study demonstrated that regular exercise has significant effects on bone metabolism in postmenopausal women by reducing bone resorption and enhancing bone formation. Aerobic exercise effectively decreases CTX levels, while both aerobic and resistance exercise effectively increase OC levels. Short-term interventions (≤6 months) and moderate-duration sessions (≤60 minutes per session) show notable benefits in lowering CTX and elevating OC. However, more rigorously designed randomized controlled trials are needed to confirm the benefits of exercise on bone metabolism and to determine the optimal intervention strategies. Due to the small number of studies, it was not possible to determine the effects on other bone metabolism metrics. However, more rigorously designed randomized controlled trials are needed to validate the benefits of exercise on bone metabolism and to explore its optimal protocol.

## Data Availability

The original contributions presented in the study are included in the article/**Supplementary Material**. Further inquiries can be directed to the corresponding author.
