# Supplementary material for: Effects of exercise on bone metabolism in postmenopausal women: a systematic review and meta-analysis of randomized controlled trials
Source: Front Endocrinol (Lausanne). 2025 Sep 15;16:1597046. doi: 10.3389/fendo.2025.1597046 (PMC12477224; doi:10.3389/fendo.2025.1597046)
Supplement: Supplementary file 1 [file DataSheet1.docx]

Supplementary Material

# Supplementary Tables and Figures

## Supplementary Tables

**Supplementary Table S1.** The Preferred Reporting Items for Systematic reviews and Meta-Analysis (PRISMA) 2020 Main Checklist.

| **Topic** | **No.** | **Item** | **Location where item is reported** |
| --- | --- | --- | --- |
| **TITLE** |  |  |  |
| **Title** | 1 | Identify the report as a systematic review. | Title |
| **ABSTRACT** |  |  |  |
| **Abstract** | 2 | See the PRISMA 2020 for Abstracts checklist | Abstract |
| **INTRODUCTION** |  |  |  |
| **Rationale** | 3 | Describe the rationale for the review in the context of existing knowledge. | 1 Introduction |
| **Objectives** | 4 | Provide an explicit statement of the objective(s) or question(s) the review addresses. | 1 Introduction |
| **METHODS** |  |  |  |
| **Eligibility criteria** | 5 | Specify the inclusion and exclusion criteria for the review and how studies were grouped for the syntheses. | 2.3 Inclusion and exclusion criteria for the studies |
| **Information sources** | 6 | Specify all databases, registers, websites, organizations, reference lists and other sources searched or consulted to identify studies. Specify the date when each source was last searched or consulted. | 2.2 Search strategy |
| **Search strategy** | 7 | Present the full search strategies for all databases, registers and websites, including any filters and limits used. | 2.2 Search strategy |
| **Selection process** | 8 | Specify the methods used to decide whether a study met the inclusion criteria of the review, including how many reviewers screened each record and each report retrieved, whether they worked independently, and if applicable, details of automation tools used in the process. | 2.4 Literature screening and data extraction |
| **Data collection process** | 9 | Specify the methods used to collect data from reports, including how many reviewers collected data from each report, whether they worked independently, any processes for obtaining or confirming data from study investigators, and if applicable, details of automation tools used in the process. | 2.4 Literature screening and data extraction |
| **Data items** | 10a | List and define all outcomes for which data were sought. Specify whether all results that were compatible with each outcome domain in each study were sought (e.g. for all measures, time points, analyses), and if not, the methods used to decide which results to collect. | 2.3 Inclusion and exclusion criteria for the studies  2.4 Literature screening and data extraction |
|  | 10b | List and define all other variables for which data were sought (e.g. participant and intervention characteristics, funding sources). Describe any assumptions made about any missing or unclear information. | 2.4 Literature screening and data extraction |
| **Study risk of bias assessment** | 11 | Specify the methods used to assess risk of bias in the included studies, including details of the tool(s) used, how many reviewers assessed each study and whether they worked independently, and if applicable, details of automation tools used in the process. | 2.5 Risk of bias assessment |
| **Effect measures** | 12 | Specify for each outcome the effect measure(s) (e.g. risk ratio, mean difference) used in the synthesis or presentation of results. | 2.6 Data analysis |
| **Synthesis methods** | 13a | Describe the processes used to decide which studies were eligible for each synthesis (e.g. tabulating the study intervention characteristics and comparing against the planned groups for each synthesis (item 5)). | TABLE 1 Research characteristics  TABLE 2 Characterization of research interventions |
|  | 13b | Describe any methods required to prepare the data for presentation or synthesis, such as handling of missing summary statistics, or data conversions. | 2.6 Data analysis |
|  | 13c | Describe any methods used to tabulate or visually display results of individual studies and syntheses. | TABLE 1 Research characteristics  TABLE 2 Characterization of research interventions |
|  | 13d | Describe any methods used to synthesize results and provide a rationale for the choice(s). If meta-analysis was performed, describe the model(s), method(s) to identify the presence and extent of statistical heterogeneity, and software package(s) used. | 2.6 Data analysis |
|  | 13e | Describe any methods used to explore possible causes of heterogeneity among study results (e.g. subgroup analysis, meta-regression). | 2.7 Subgroup analysis |
|  | 13f | Describe any sensitivity analyses conducted to assess robustness of the synthesized results. | 2.8 Sensitivity analysis |
| **Reporting bias assessment** | 14 | Describe any methods used to assess risk of bias due to missing results in a synthesis (arising from reporting biases). | 2.5 Risk of bias assessment |
| **Certainty assessment** | 15 | Describe any methods used to assess certainty (or confidence) in the body of evidence for an outcome. | 2.10 Certainty of evidence |
| **RESULTS** |  |  |  |
| **Study selection** | 16a | Describe the results of the search and selection process, from the number of records identified in the search to the number of studies included in the review, ideally using a flow diagram. | 3.1 Results of literature screening  FIGURE 1  PRISMA study flow diagram. |
|  | 16b | Cite studies that might appear to meet the inclusion criteria, but which were excluded, and explain why they were excluded. | 3.1 Results of literature screening |
| **Study characteristics** | 17 | Cite each included study and present its characteristics. | 3.2 Characteristics of the included studies |
| **Risk of bias in studies** | 18 | Present assessments of risk of bias for each included study. | 3.3 Risk of bias |
| **Results of individual studies** | 19 | For all outcomes, present, for each study: (a) summary statistics for each group (where appropriate) and (b) an effect estimates and its precision (e.g. confidence/credible interval), ideally using structured tables or plots. | 3.4 Meta-analysis results |
| **Results of syntheses** | 20a | For each synthesis, briefly summaries the characteristics and risk of bias among contributing studies. | 3.3 Risk of bias  3.4 Meta-analysis results |
|  | 20b | Present results of all statistical syntheses conducted. If meta-analysis was done, present for each the summary estimate and its precision (e.g. confidence/credible interval) and measures of statistical heterogeneity. If comparing groups, describe the direction of the effect. | 3.4 Meta-analysis results |
|  | 20c | Present results of all investigations of possible causes of heterogeneity among study results. | 3.4 Meta-analysis results |
|  | 20d | Present results of all sensitivity analyses conducted to assess the robustness of the synthesized results. | 3.5 Sensitivity analysis |
| **Reporting biases** | 21 | Present assessments of risk of bias due to missing results (arising from reporting biases) for each synthesis assessed. | - |
| **Certainty of evidence** | 22 | Present assessments of certainty (or confidence) in the body of evidence for each outcome assessed. | 3.6 Certainty of evidence |
| **DISCUSSION** |  |  |  |
| **Discussion** | 23a | Provide a general interpretation of the results in the context of other evidence. | 4 Discussion |
|  | 23b | Discuss any limitations of the evidence included in the review. | 4 Discussion |
|  | 23c | Discuss any limitations of the review processes used. | 4 Discussion |
|  | 23d | Discuss implications of the results for practice, policy, and future research. | 4 Discussion |
| **OTHER INFORMATION** |  |  |  |
| **Registration and protocol** | 24a | Provide registration information for the review, including register name and registration number, or state that the review was not registered. | 2.1 Protocol and registration |
|  | 24b | Indicate where the review protocol can be accessed, or state that a protocol was not prepared. | 2.1 Protocol and registration |
|  | 24c | Describe and explain any amendments to information provided at registration or in the protocol. | 2.1 Protocol and registration |
| **Support** | 25 | Describe sources of financial or non-financial support for the review, and the role of the funders or sponsors in the review. | Funding |
| **Competing interests** | 26 | Declare any competing interests of review authors. | Conflict of interest |
| **Availability of data, code and other materials** | 27 | Report which of the following are publicly available and where they can be found template data collection forms; data extracted from included studies; data used for all analyses; analytic code; any other materials used in the review. | Data availability statement |

**Supplementary Table S2.** Database search strategy.

| **Database** | Search strategies |
| --- | --- |
| **PubMed** | ((((((((((((((((((Osteoporosis, Post-Menopausal[MeSH]) OR (Osteoporoses, Post-Menopausal[Title/Abstract])) OR (Osteoporosis, Post Menopausal[Title/Abstract])) OR (Post-Menopausal Osteoporoses[Title/Abstract])) OR (Post-Menopausal Osteoporosis[Title/Abstract])) OR (Postmenopausal Osteoporosis[Title/Abstract])) OR (Osteoporoses, Postmenopausal)) OR (Postmenopausal Osteoporoses[Title/Abstract])) OR (Perimenopausal Bone Loss[Title/Abstract])) OR (Bone Loss, Postmenopausal[Title/Abstract])) OR (Bone Losses, Postmenopausal[Title/Abstract])) OR (Postmenopausal Bone Losses[Title/Abstract])) OR (Postmenopausal Bone Loss[Title/Abstract])) OR (Bone Loss, Perimenopausal[Title/Abstract])) OR (Bone Losses, Perimenopausal[Title/Abstract])) OR (Perimenopausal Bone Losses[Title/Abstract])) AND (((((((((((((((((((((((((Exercises[MeSH]) OR (Exercise, Physical[Title/Abstract])) OR (Exercises, Physical[Title/Abstract])) OR (Physical Exercise[Title/Abstract])) OR (Physical Exercises[Title/Abstract])) OR (Exercise, Aerobic[Title/Abstract])) OR (Aerobic Exercise[Title/Abstract])) OR (Aerobic Exercises[Title/Abstract])) OR (Exercises, Aerobic[Title/Abstract])) OR (Exercise, Isometric)) OR (Exercises, Isometric)) OR (Isometric Exercises[Title/Abstract])) OR (Isometric Exercise[Title/Abstract])) OR (Acute Exercise[Title/Abstract])) OR (Acute Exercises[Title/Abstract])) OR (Exercise, Acute[Title/Abstract])) OR (Exercises, Acute[Title/Abstract])) OR (Exercise Training[Title/Abstract])) OR (Exercise Trainings[Title/Abstract])) OR (Training, Exercise[Title/Abstract])) OR (Trainings, Exercise[Title/Abstract])) OR (Physical Activity[Title/Abstract])) OR (Activities, Physical[Title/Abstract])) OR (Activity, Physical[Title/Abstract])) OR (Physical Activities[Title/Abstract]))) AND (((Randomized controlled trial[MeSH]) OR (Randomly[Title/Abstract])) OR (trial[Title/Abstract]))) AND (((osteometabolism[MeSH]) OR (bone metabolism[Title/Abstract])) OR (osteo-metabolism[Title/Abstract])) |
| **Embase** | 'osteoporosis, post-menopausal'/de OR 'osteoporoses, post-menopausal':ti,ab OR 'osteoporosis, post-menopausal':ti,ab OR 'post-menopausal osteoporoses':ti,ab OR 'post-menopausal osteoporosis':ti,ab OR 'postmenopausal osteoporosis':ti,ab OR 'osteoporoses, postmenopausal':ti,ab OR 'postmenopausal osteoporoses':ti,ab OR 'perimenopausal bone loss':ti,ab OR 'bone loss, postmenopausal':ti,ab OR 'bone losses, postmenopausal':ti,ab OR 'postmenopausal bone losses':ti,ab OR 'postmenopausal bone loss':ti,ab OR 'bone loss, perimenopausal':ti,ab OR 'bone losses, perimenopausal':ti,ab OR 'perimenopausal bone losses':ti,ab AND 'exercises'/de OR 'exercise, physical':ti,ab OR 'exercises, physical':ti,ab OR 'physical exercise':ti,ab OR 'physical exercises':ti,ab OR 'exercise, aerobic':ti,ab OR 'aerobic exercise':ti,ab OR 'aerobic exercises':ti,ab OR 'exercises, aerobic':ti,ab OR 'exercise, isometric':ti,ab OR 'exercises, isometric':ti,ab OR 'isometric exercises':ti,ab OR 'isometric exercise':ti,ab OR 'acute exercise':ti,ab OR 'acute exercises':ti,ab OR 'exercise, acute':ti,ab OR 'exercises, acute':ti,ab OR 'exercise training':ti,ab OR 'exercise trainings':ti,ab OR 'training, exercise':ti,ab OR 'trainings, exercise':ti,ab OR 'physical activity':ti,ab OR 'activities, physical':ti,ab OR 'activity, physical':ti,ab OR 'physical activities':ti,ab AND 'randomized controlled trial'/de OR 'randomly':ti,ab OR 'trial':ti,ab AND 'osteometabolism'/de OR 'bone metabolism':ti,ab OR 'osteo-metabolism':ti,ab |
| **Cochrane library** | (osteoporosis, post-menopausal[MeSH] OR osteoporoses, post-menopausal[tiab] OR osteoporosis, post-menopausal[tiab] OR post-menopausal osteoporoses[tiab] OR post-menopausal osteoporosis[tiab] OR postmenopausal osteoporosis[tiab] OR osteoporoses, postmenopausal[tiab] OR postmenopausal osteoporoses[tiab] OR perimenopausal bone loss[tiab] OR bone loss, postmenopausal[tiab] OR bone losses, postmenopausal[tiab] OR postmenopausal bone losses[tiab] OR postmenopausal bone loss[tiab] OR bone loss, perimenopausal[tiab] OR bone losses, perimenopausal[tiab] OR perimenopausal bone losses[tiab])  AND  (exercises[MeSH] OR exercise, physical[tiab] OR exercises, physical[tiab] OR physical exercise[tiab] OR physical exercises[tiab] OR exercise, aerobic[tiab] OR aerobic exercise[tiab] OR aerobic exercises[tiab] OR exercises, aerobic[tiab] OR exercise, isometric[tiab] OR exercises, isometric[tiab] OR isometric exercises[tiab] OR isometric exercise[tiab] OR acute exercise[tiab] OR acute exercises[tiab] OR exercise, acute[tiab] OR exercises, acute[tiab] OR exercise training[tiab] OR exercise trainings[tiab] OR training, exercise[tiab] OR trainings, exercise[tiab] OR physical activity[tiab] OR activities, physical[tiab] OR activity, physical[tiab] OR physical activities[tiab])  AND  (randomized controlled trial [MeSH] OR randomly[tiab] OR trial[tiab])  AND  (osteometabolism [MeSH] OR bone metabolism[tiab] OR osteo-metabolism[tiab]) |
| **Web of Science** | TS=(("Osteoporosis, Post-Menopausal" OR "Osteoporoses, Post-Menopausal" OR "Osteoporosis, Post Menopausal" OR "Post-Menopausal Osteoporoses" OR "Post-Menopausal Osteoporosis" OR "Postmenopausal Osteoporosis" OR "Osteoporoses, Postmenopausal" OR "Postmenopausal Osteoporoses" OR "Perimenopausal Bone Loss" OR "Bone Loss, Postmenopausal" OR "Bone Losses, Postmenopausal" OR "Postmenopausal Bone Losses" OR "Postmenopausal Bone Loss" OR "Bone Loss, Perimenopausal" OR "Bone Losses, Perimenopausal" OR "Perimenopausal Bone Losses"))  AND  TS= (("Exercises" OR "Exercise, Physical" OR "Exercises, Physical" OR "Physical Exercise" OR "Physical Exercises" OR "Exercise, Aerobic" OR "Aerobic Exercise" OR "Aerobic Exercises" OR "Exercises, Aerobic" OR "Exercise, Isometric" OR "Exercises, Isometric" OR "Isometric Exercises" OR "Isometric Exercise" OR "Acute Exercise" OR "Acute Exercises" OR "Exercise, Acute" OR "Exercises, Acute" OR "Exercise Training" OR "Exercise Trainings" OR "Training, Exercise" OR "Trainings, Exercise" OR "Physical Activity" OR "Activities, Physical" OR "Activity, Physical" OR "Physical Activities"))  AND  TS= (("Randomized controlled trial" OR "Randomly" OR "trial")  )  AND  TS= (("osteometabolism" OR "bone metabolism" OR "osteo-metabolism" ) |
| **Scopus** | (TITLE-ABS-KEY("post-menopausal osteoporosis") OR TITLE-ABS-KEY("osteoporoses, post-menopausal") OR TITLE-ABS-KEY("osteoporosis, post-menopausal") OR TITLE-ABS-KEY("post-menopausal osteoporoses") OR TITLE-ABS-KEY("postmenopausal osteoporosis") OR TITLE-ABS-KEY("osteoporoses, postmenopausal") OR TITLE-ABS-KEY("postmenopausal osteoporoses") OR TITLE-ABS-KEY("perimenopausal bone loss") OR TITLE-ABS-KEY("bone loss, postmenopausal") OR TITLE-ABS-KEY("bone losses, postmenopausal") OR TITLE-ABS-KEY("postmenopausal bone losses") OR TITLE-ABS-KEY("postmenopausal bone loss") OR TITLE-ABS-KEY("bone loss, perimenopausal") OR TITLE-ABS-KEY("bone losses, perimenopausal") OR TITLE-ABS-KEY("perimenopausal bone losses")) AND (TITLE-ABS-KEY("exercises") OR TITLE-ABS-KEY("exercise, physical") OR TITLE-ABS-KEY("physical exercise") OR TITLE-ABS-KEY("physical exercises") OR TITLE-ABS-KEY("exercise, aerobic") OR TITLE-ABS-KEY("aerobic exercise") OR TITLE-ABS-KEY("aerobic exercises") OR TITLE-ABS-KEY("exercises, aerobic") OR TITLE-ABS-KEY("exercise, isometric") OR TITLE-ABS-KEY("exercises, isometric") OR TITLE-ABS-KEY("isometric exercises") OR TITLE-ABS-KEY("isometric exercise") OR TITLE-ABS-KEY("acute exercise") OR TITLE-ABS-KEY("acute exercises") OR TITLE-ABS-KEY("exercise, acute") OR TITLE-ABS-KEY("exercises, acute") OR TITLE-ABS-KEY("exercise training") OR TITLE-ABS-KEY("exercise trainings") OR TITLE-ABS-KEY("training, exercise") OR TITLE-ABS-KEY("trainings, exercise") OR TITLE-ABS-KEY("physical activity") OR TITLE-ABS-KEY("activities, physical") OR TITLE-ABS-KEY("activity, physical") OR TITLE-ABS-KEY("physical activities")) AND (TITLE-ABS-KEY("randomized controlled trial") OR TITLE-ABS-KEY("randomly") OR TITLE-ABS-KEY("trial")) AND (TITLE-ABS-KEY("osteometabolism") OR TITLE-ABS-KEY("bone metabolism") OR TITLE-ABS-KEY("osteo-metabolism")) |
| **Google Scholar** | ("Osteoporosis, Post-Menopausal" OR "Osteoporoses, Post-Menopausal" OR "Osteoporosis, Post Menopausal" OR "Post-Menopausal Osteoporoses" OR "Post-Menopausal Osteoporosis" OR "Postmenopausal Osteoporosis" OR "Osteoporoses, Postmenopausal" OR "Postmenopausal Osteoporoses" OR "Perimenopausal Bone Loss" OR "Bone Loss, Postmenopausal" OR "Bone Losses, Postmenopausal" OR "Postmenopausal Bone Losses" OR "Postmenopausal Bone Loss" OR "Bone Loss, Perimenopausal" OR "Bone Losses, Perimenopausal" OR "Perimenopausal Bone Losses") AND ("Exercises" OR "Exercise, Physical" OR "Exercises, Physical" OR "Physical Exercise" OR "Physical Exercises" OR "Exercise, Aerobic" OR "Aerobic Exercise" OR "Aerobic Exercises" OR "Exercises, Aerobic" OR "Exercise, Isometric" OR "Exercises, Isometric" OR "Isometric Exercises" OR "Isometric Exercise" OR "Acute Exercise" OR "Acute Exercises" OR "Exercise, Acute" OR "Exercises, Acute" OR "Exercise Training" OR "Exercise Trainings" OR "Training, Exercise" OR "Trainings, Exercise" OR "Physical Activity" OR "Activities, Physical" OR "Activity, Physical" OR "Physical Activities") AND ("Randomized controlled trial" OR "Randomly" OR "trial") AND ("osteometabolism" OR "bone metabolism" OR "osteo-metabolism") |

**Supplementary Table S3. Certainy of evidence.**

**Author(s):** Wenhua Zhang

**Question:** [exercise] compared to [no exercise] for [bone metabolism in postmenopausal women]

**Bibliography:** Zhang Y, Akl EA, Schünemann HJ. Using systematic reviews in guideline development: the GRADE approach. Res Synth Methods. 2019;10(3).

| **Certainty assessment** | | | | | | | **№ of patients** | | **Effect** | | **Certainty** | **Importance** |
| --- | --- | --- | --- | --- | --- | --- | --- | --- | --- | --- | --- | --- |
| **№ of studies** | **Study design** | **Risk of bias** | **Inconsistency** | **Indirectness** | **Imprecision** | **Other considerations** | **[exercise]** | **[no-exercise]** | **Relative (95% CI)** | **Absolute (95% CI)** |  |  |
| **serum phosphorus** | | | | | | | | | | | | |
| 4 | randomised trials | very serious^a,b,c^ | not serious | not serious | serious^d^ | none | 122 | 82 | - | SMD **0.1 SD higher** (0.18 lower to 0.39 higher) | ⨁◯◯◯ Very low^a,b,c,d^ |  |
| **serum calcium** | | | | | | | | | | | | |
| 5 | randomised trials | very serious^a,b,c^ | not serious | not serious | serious^d^ | none | 181 | 123 | - | SMD **0.1 SD higher** (0.13 lower to 0.34 higher) | ⨁◯◯◯ Very low^a,b,c,d^ |  |
| **serum 25(OH)D** | | | | | | | | | | | | |
| 5 | randomised trials | very serious^a,b,c^ | not serious | not serious | serious^d^ | none | 190 | 131 | - | SMD **0.18 SD higher** (0.04 lower to 0.41 higher) | ⨁◯◯◯ Very low^a,b,c,d^ |  |
| **serum PTH** | | | | | | | | | | | | |
| 5 | randomised trials | very serious^a,b,c^ | not serious | not serious | serious^d^ | none | 157 | 102 | - | SMD **0.51 SD lower** (0.77 lower to 0.25 lower) | ⨁◯◯◯ Very low^a,b,c,d^ |  |
| **serum ALP** | | | | | | | | | | | | |
| 5 | randomised trials | very serious^a,b,c^ | not serious | not serious | serious^d^ | none | 112 | 99 | - | SMD **0.49 SD higher** (0.21 higher to 0.77 higher) | ⨁◯◯◯ Very low^a,b,c,d^ |  |
| **serum P1NP** | | | | | | | | | | | | |
| 3 | randomised trials | very serious^a,b,c^ | not serious | not serious | serious^d^ | none | 63 | 51 | - | SMD **0.62 SD higher** (0.24 higher to 1.01 higher) | ⨁◯◯◯ Very low^a,b,c,d^ |  |
| **serum CTX** | | | | | | | | | | | | |
| 7 | randomised trials | very serious^a,b,c^ | not serious | not serious | not serious | none | 266 | 180 | - | SMD **0.32 SD lower** (0.51 lower to 0.12 lower) | ⨁⨁◯◯ Low^a,b,c^ |  |
| **serum OC** | | | | | | | | | | | | |
| 11 | randomised trials | very serious^a,b,c^ | not serious | not serious | not serious | none | 388 | 277 | - | SMD **0.21 SD higher** (0.05 higher to 0.37 higher) | ⨁⨁◯◯ Low^a,b,c^ |  |

**CI:** confidence interval; **SMD:** standardised mean difference; **OC**=osteocalcin; **PTH**=parathyroid hormone; **ALP**=alkaline phosphatase; **25(OH)D**=25-hydroxyvitamin D; **CTX**=type I collagen cross-linked C-terminal peptide; **P1NP**=N-terminal propeptide of type I procollagen

#### Explanations

a. Some studies did not provide specific randomization methods

b. Don't blind researchers and participants in experiments or provided insufficient information

c. Provide insufficient information on blinding of outcome assessment or high risk for blinding of outcome assessment

d. The sample size is low

## Supplementary Figures


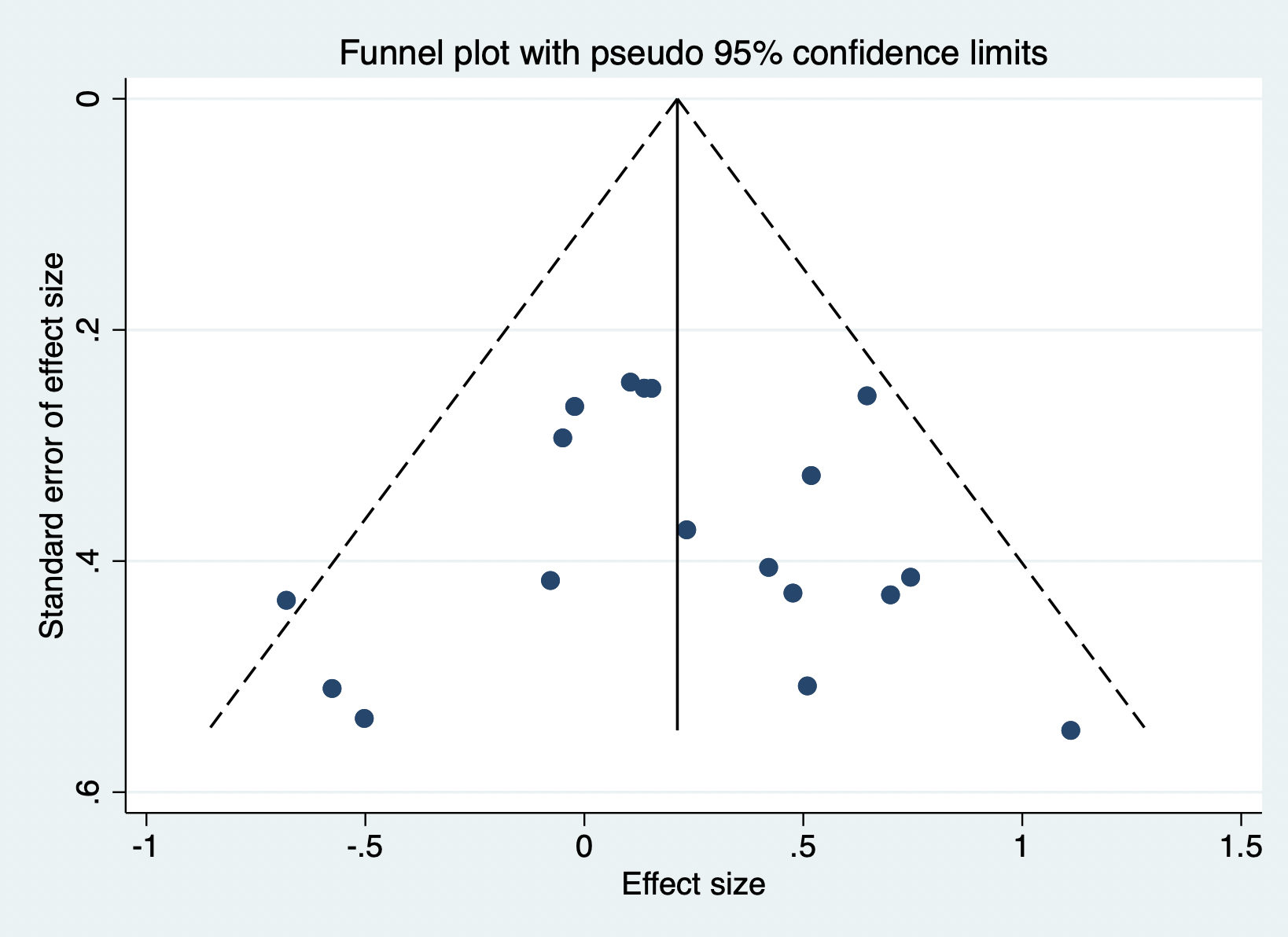


**Figure S1.** Funnel plot of the effect of exercise on osteocalcin in postmenopausal women.


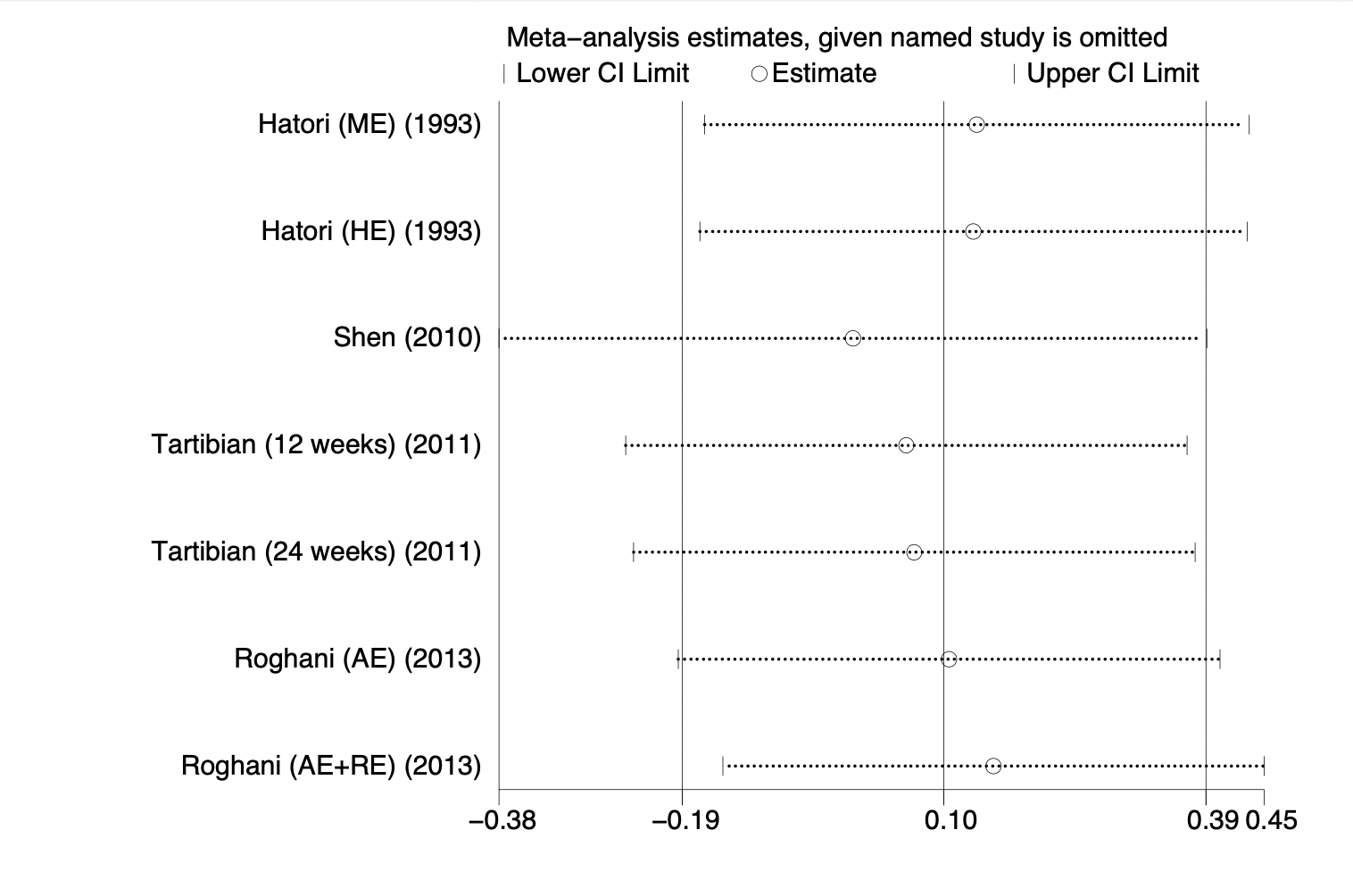


**Supplementary Figure S2.** Sensitivity analyses completed by the metaninf command (serum phosphorus).

Abbreviations: CI=confidence interval; HE= high intensity exercise; ME=moderate intensity exercise; AE=aerobic exercise; RE=resistance exercise.


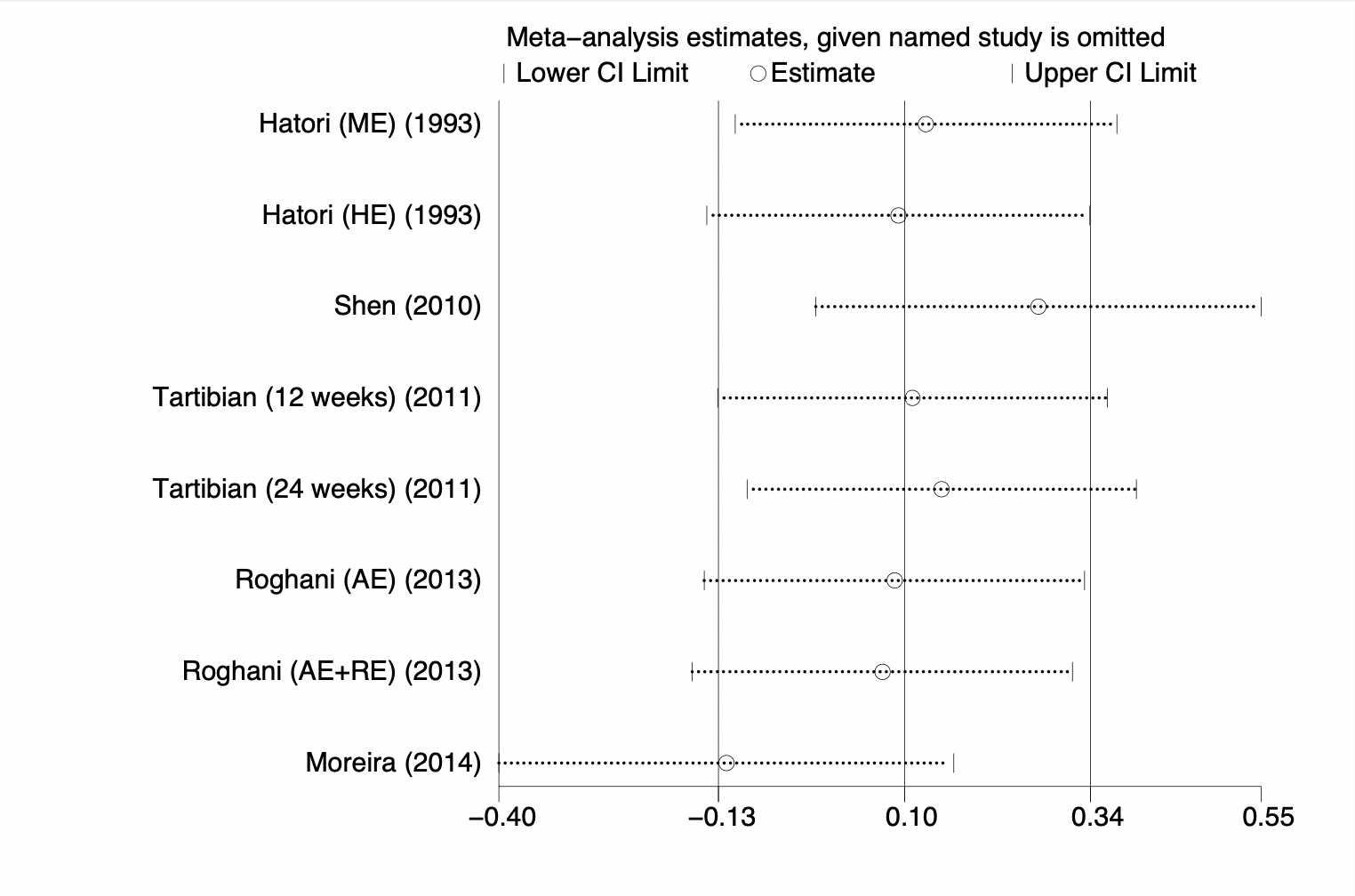


**Supplementary Figure S3.** Sensitivity analyses completed by the metaninf command (serum calcium).

Abbreviations: CI=confidence interval; HE= high intensity exercise; ME=moderate intensity exercise; AE=aerobic exercise; RE=resistance exercise.

**
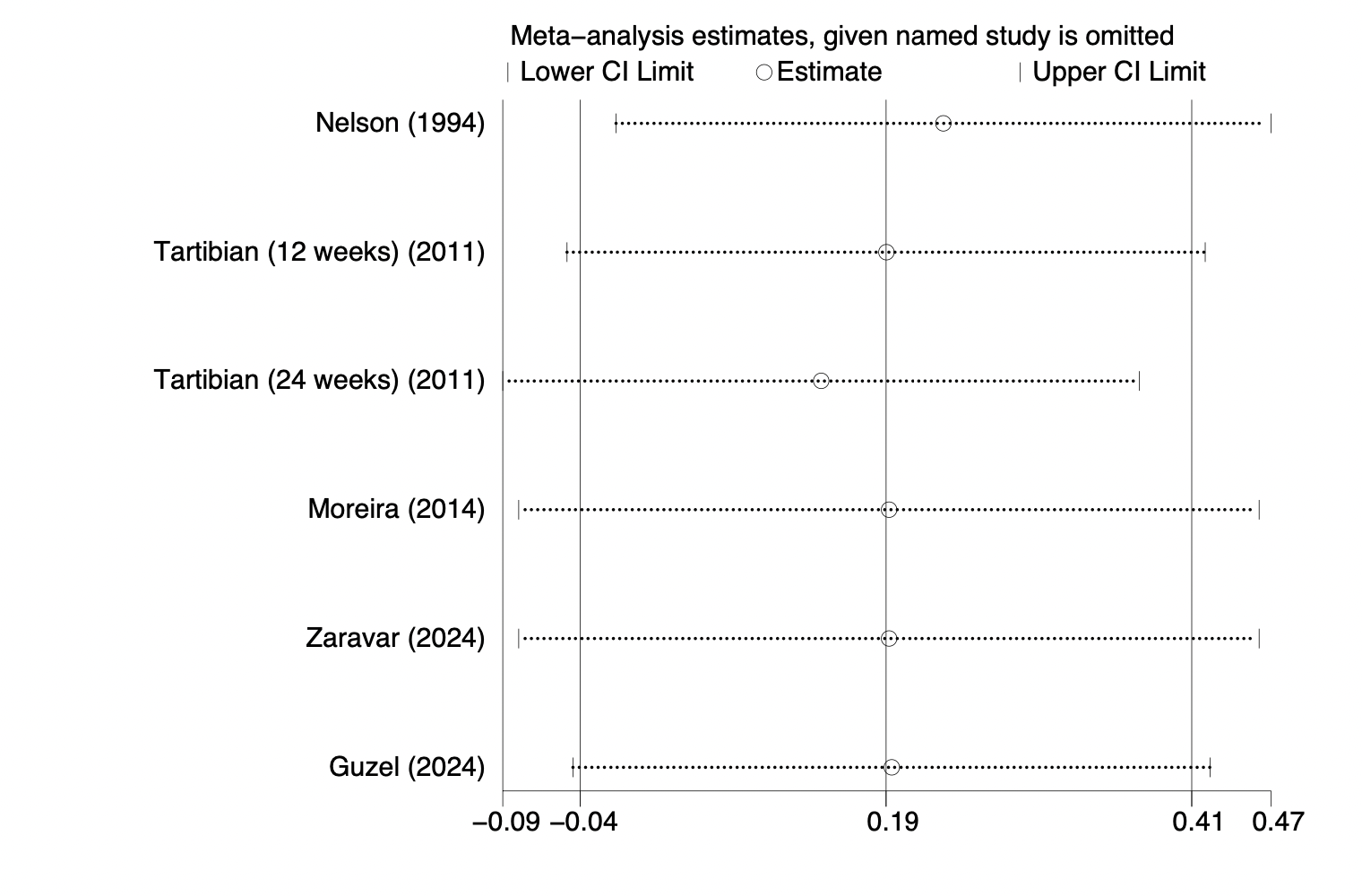
**

**Supplementary Figure S4.** Sensitivity analyses completed by the metaninf command [25(OH)D].

Abbreviations: CI=confidence interval; 25(OH)D=25-hydroxyvitamin D.


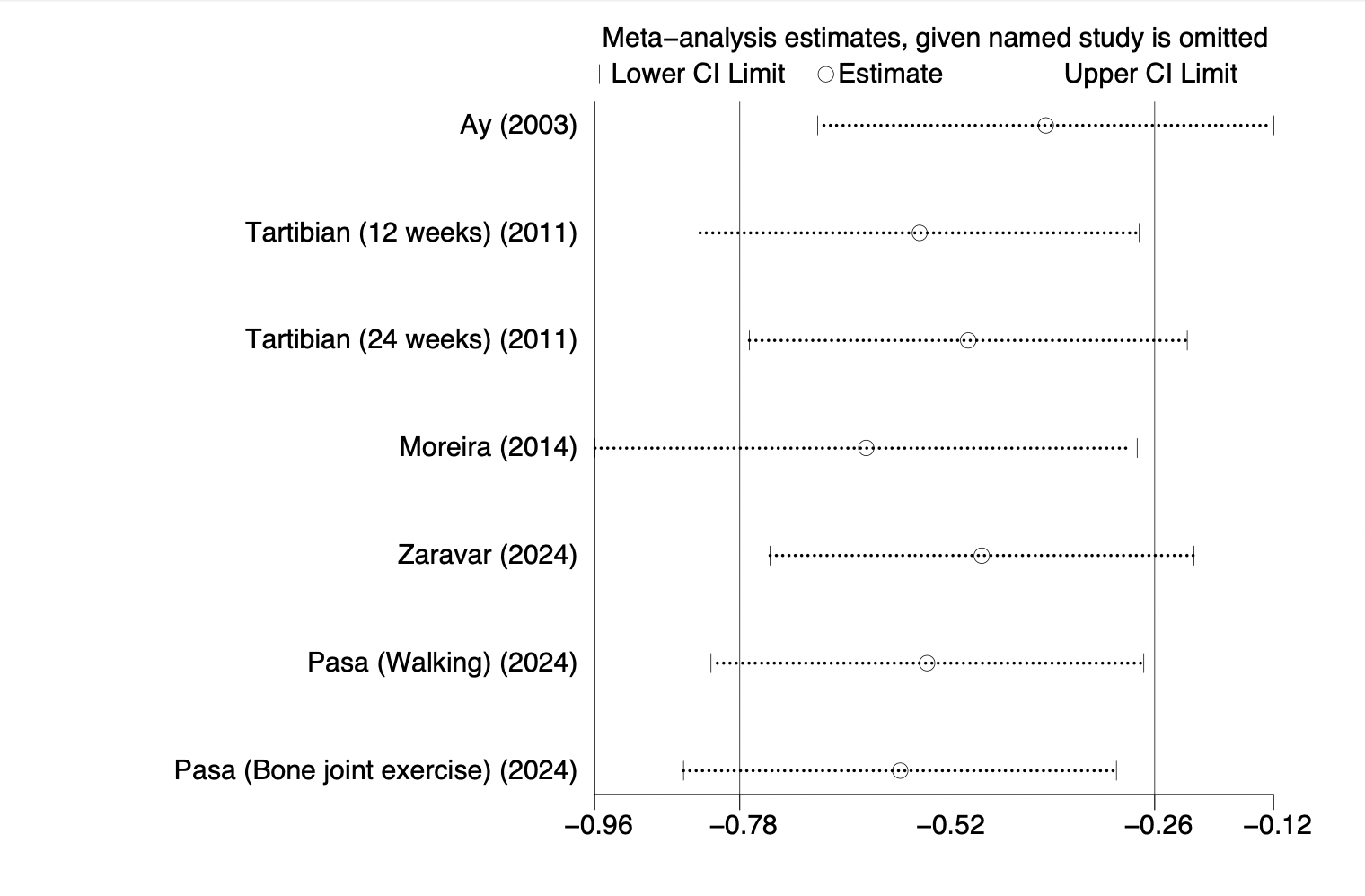


**Supplementary Figure S5.** Sensitivity analyses completed by the metaninf command (PTH).

Abbreviations: CI=confidence interval; PTH=parathyroid hormone.


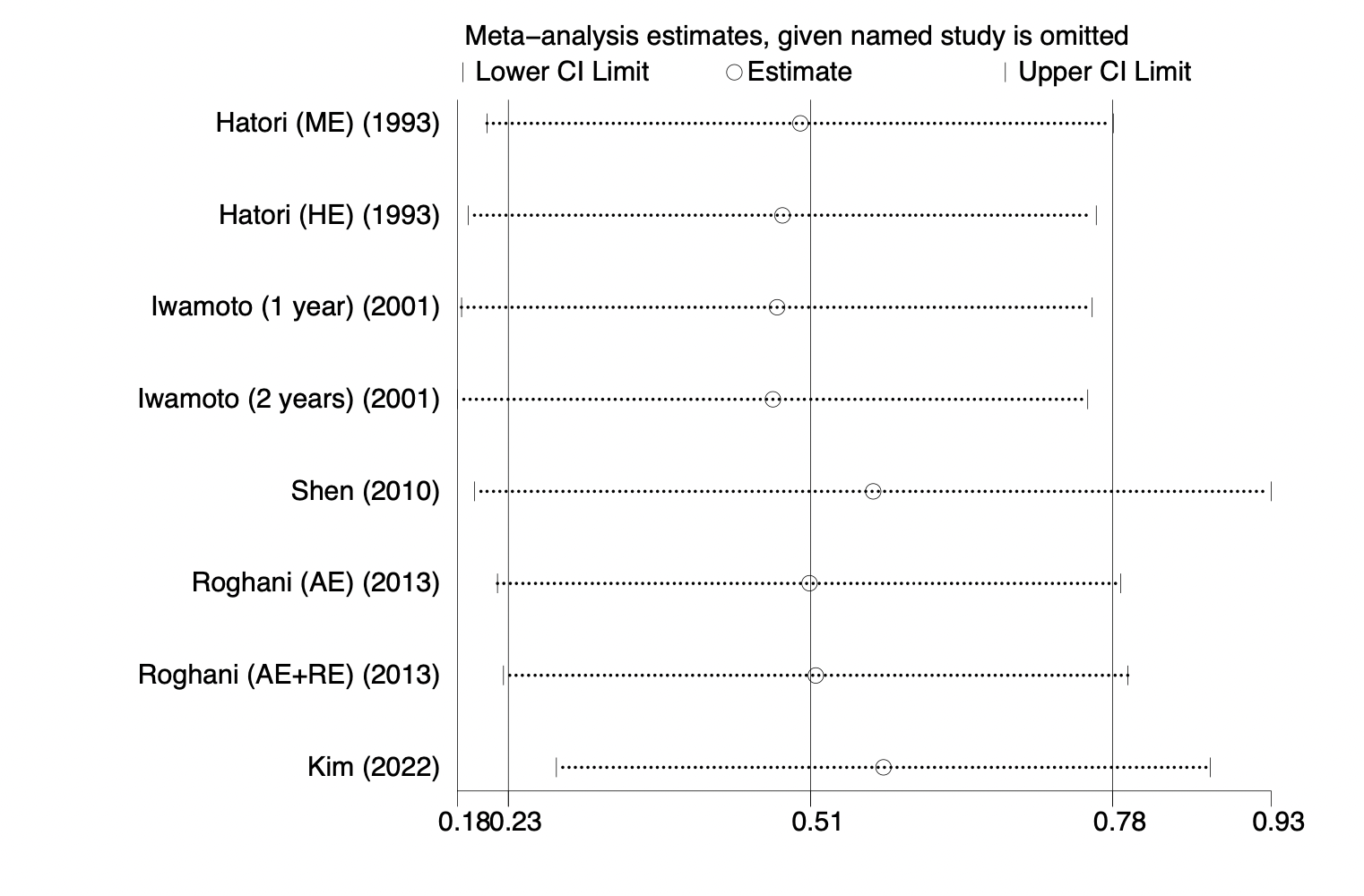


**Supplementary Figure S6.** Sensitivity analyses completed by the metaninf command (ALP).

Abbreviations: CI=confidence interval; HE= high intensity exercise; ME=moderate intensity exercise; AE=aerobic exercise; RE=resistance exercise; ALP=alkaline phosphatase.


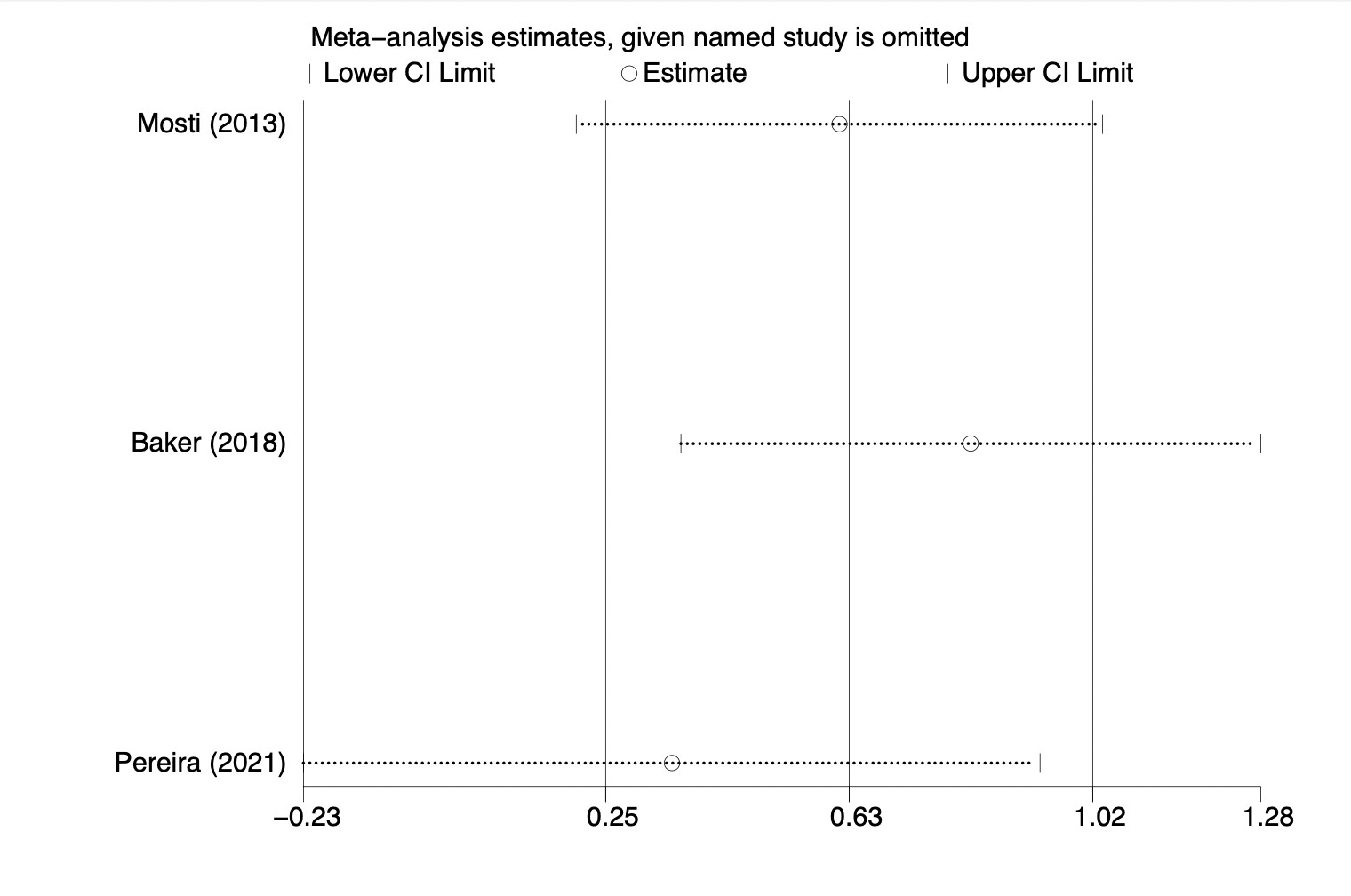


**Supplementary Figure S7.** Sensitivity analyses completed by the metaninf command (P1NP).

Abbreviations: CI=confidence interval; P1NP=N-terminal propeptide of type I procollagen.


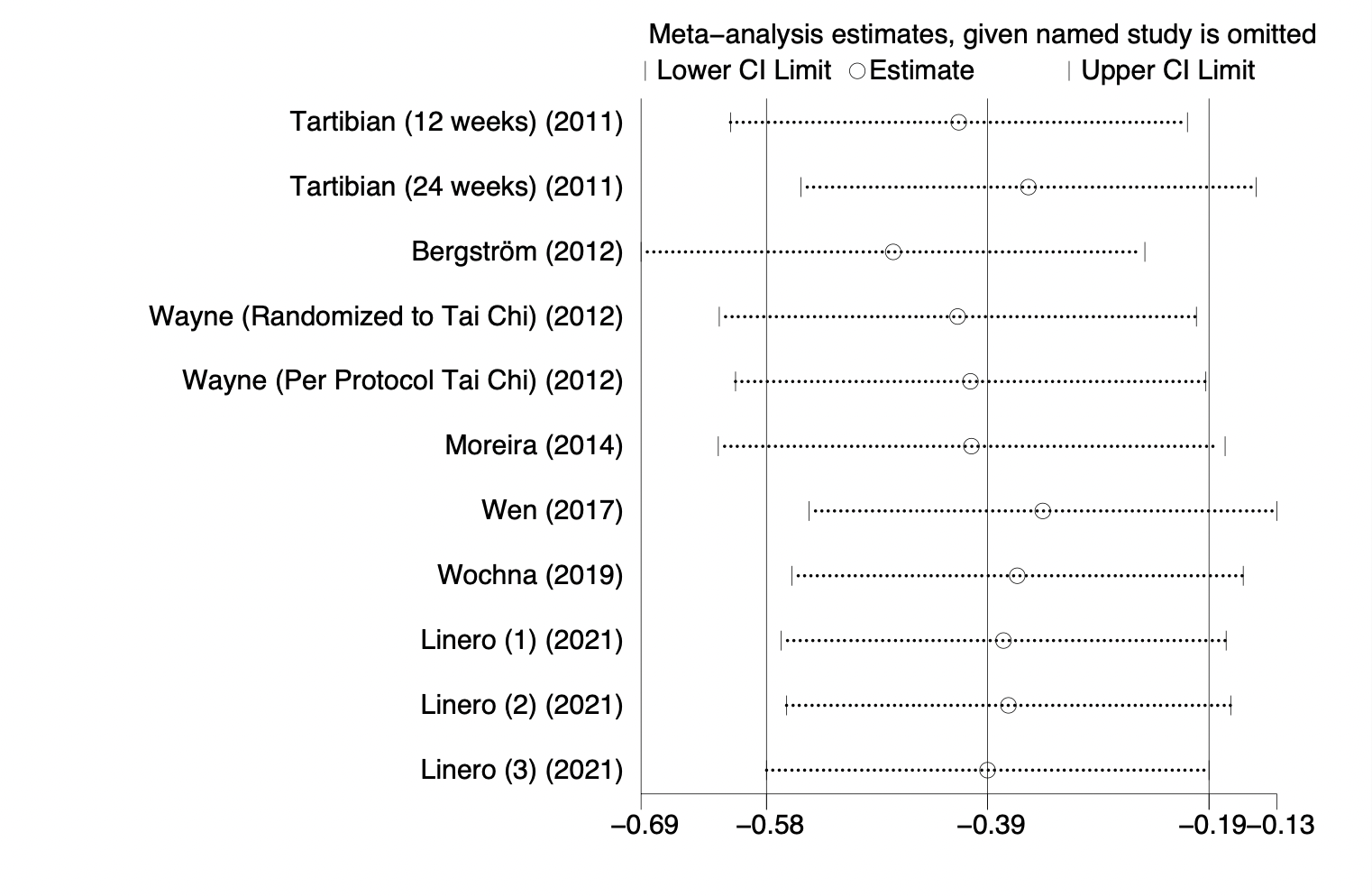


**Supplementary Figure S8.** Sensitivity analyses completed by the metaninf command (CTX).

Abbreviations: CI=confidence interval; CTX=type I collagen cross-linked C-terminal peptide.


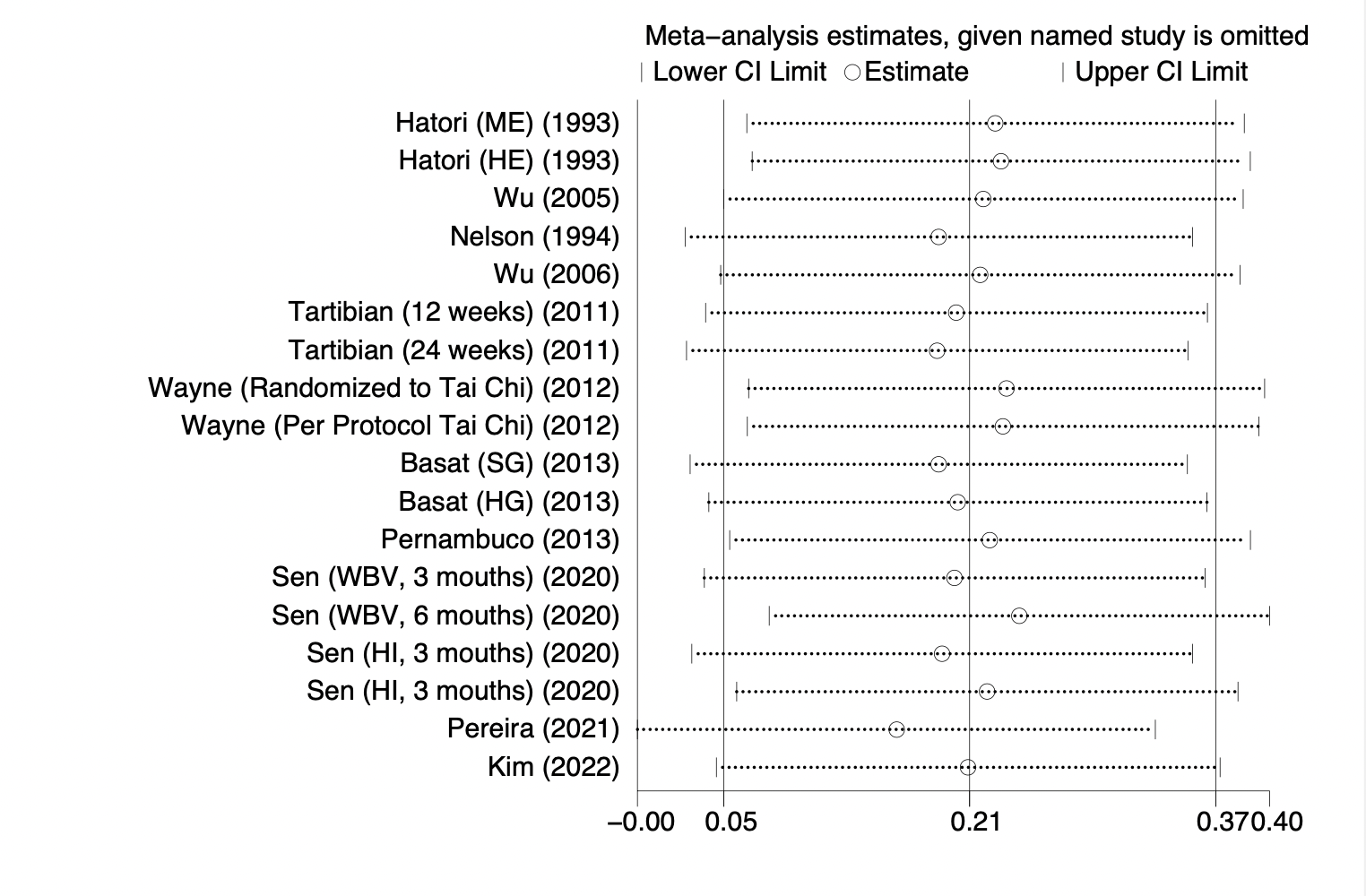


**Supplementary Figure S9.** Sensitivity analyses completed by the metaninf command (OC).

Abbreviations: CI=confidence interval; HE= high intensity exercise; ME=moderate intensity exercise; SG=strength training group; HG= high intensity impact training group; WBV= whole body vibration; HI= high impact; OC=osteocalcin.
